# Impact of abdominal obesity prevalence trends on dementia, cardiovascular disease, functional impairment, and mortality in older Chinese adults: A Markov scenario simulation, 2020–2050

**DOI:** 10.1371/journal.pmed.1004970

**Published:** 2026-04-07

**Authors:** Yujing Zhou, Minrui Zeng, Yuntao Chen, Piotr Bandosz, Eric Brunner, Jing Liao

**Affiliations:** 1 Department of Medical Statistics, School of Public Health, Sun Yat-sen University, Guangzhou, China; 2 Guangzhou Baiyun International Airport Co., Ltd, Guangzhou, China; 3 Department of Epidemiology & Public Health, University College London, London, United Kingdom; 4 Department of Public Health and Policy, University of Liverpool, Liverpool, United Kingdom; 5 Department of Prevention and Medical Education, Medical University of Gdansk, Gdańsk, Poland; 6 Sun Yat-sen Global Health Institute, Institute of State Governance, Sun Yat-sen University, Guangzhou, China; Waymark, UNITED STATES OF AMERICA

## Abstract

**Background:**

Dementia, cardiovascular disease (CVD), and functional impairment (FI) often co-occur in aging populations, with abdominal obesity as a shared modifiable risk factor. The long-term impact of abdominal obesity on these comorbidities is unclear. We projected the 30-year burden of dementia, FI, and CVD in China under different trajectories of abdominal obesity prevalence.

**Methods and findings:**

We modeled three trajectories of abdominal obesity prevalence from 2020 to 2050 using data from the China Health and Nutrition Survey (2000–2015): continuation of the observed growth prevalence trend (persistent), stabilization at 2015 levels (optimal), and a 50% reduction in the growth rate (improved). Abdominal obesity was defined as a waist circumference of ≥90 cm for men and ≥85 cm for women. A Markov model was used to estimate occurrence of dementia, CVD, FI, and mortality among adults aged ≥65 years by sex and year.

Under the persistent scenario, dementia cases were projected to rise to 37.8 million (95% uncertainty interval (UI) [36.7, 38.9]) by 2050, alongside 68.1 million (95% UI [66.7, 69.5]) cases of FI, 198.3 million (95% UI [196.5, 200.4]) CVD cases, and 17.6 million (95% UI [16.5, 18.7]) deaths. Compared with the persistent scenario, dementia and FI burdens increased under the optimal and improved scenarios by 2050, by 1396.0 thousand (95% UI [589.1, 2293.9]) and 711.4 thousand (95% UI [294.4, 1150.3]) for dementia, and by 2570.6 thousand (95% UI [1081.0, 4024.9]) and 1289.5 thousand (95% UI [569.5, 2034.2]) for FI, mainly due to reduced CVD mortality expanding the population at risk. These shifts are most pronounced among adults aged ≥80 years and women. For CVD, reductions in the number of cases were projected in the short term (by 2030), but these changes remain uncertain by 2050. Main limitations include the assumption that other risk factors remain unchanged, and the lack of modeling of multiple co-occurring dementia’s risk factors.

**Conclusions:**

Abdominal obesity control may reduce CVD incidence and mortality, thereby shifting the disease burden toward dementia and FI due to increased longevity, highlighting the need for integrated, life-course public health strategies responsive to the patterns of dementia and its comorbidity in older people.

## Author summary

**Why was this study done?:**

- Dementia, cardiovascular disease (CVD), and functional impairment (FI) often occur in later life, and abdominal obesity is a shared risk factor.
- Most existing studies focus on single-disease outcomes and do not account for co-occurrence or competing risks among these conditions.
- Research that reflects the demographic and epidemiological characteristics of China, including its high prevalence of abdominal obesity, remains limited.

**What did the researchers do and find?:**

- Three future abdominal obesity scenarios were generated using Chinese population representative cohort.
- A validated Markov model was applied to estimate the effects of these scenarios on dementia, CVD, FI, and mortality among adults aged 65 years or older from 2020 to 2050, stratified by sex and year.
- Simultaneous modeling of multiple disease transitions indicated that abdominal obesity reduction may shift the disease burden from CVD toward dementia and FI, with the strongest effects observed among adults aged ≥80 years and among women.

**What do these findings mean?:**

- By reducing mortality and extending life, obesity control may increase the risks of dementia and FI, resulting in a shift from CVD to dementia and FI rather than a reduction in overall disease burden.
- This shift highlights the limitations of single-disease modeling and the need for integrated life-course strategies that account for the interplay between chronic conditions and population aging.
- Public health policies should incorporate age- and sex-specific approaches to effectively support healthy longevity and manage the rising challenge of dementia and its comorbidity in older adults.
- These findings are based on modeling assumptions and focus mainly on abdominal obesity, so they may not fully capture the combined effects of multiple risk factors that influence dementia and healthy aging.

## Abstract 3

Yujing Zhou and colleagues project the 30-year burden of dementia, functional impairment, and cardiovascular disease—often co-occurring in aging populations—in China under different prevalence trajectories of abdominal obesity, a shared modifiable risk factor.

## Introduction

China’s rapidly aging population has heightened concerns about a looming dementia crisis and its associated comorbidities. More than 16 million older adults were living with dementia in 2020 [1], a number projected to rise to 66.3 million by 2050 if dementia incidence continues to increase [2]. Cardiovascular disease (CVD) increases the risk of dementia [3], and both CVD [4,5] and cognitive impairment (CI) [6] accelerate the progression of functional impairment (FI). The co-occurrence of dementia, CVD, and FI, is expected to rise substantially, leading to reduced quality of life and increased healthcare expenditure for older adults [7].

Midlife obesity is a shared modifiable risk factor for dementia [8], CVD [9] and FI [10], while the extent to which obesity simultaneously influences the development and progression of these comorbidities remains understudied. A limited number of simulation studies project future burdens of dementia [11,12] and CVD [13] under different obesity intervention scenarios, but most have focused on single-disease outcomes. Moreover, reductions in obesity-related mortality especially from CVD, may paradoxically increase the population at risk of developing dementia and FI in later life [14]. To evaluate the population-level impact of obesity control, models that jointly simulate transitions between multiple disease states and mortality are required.

Evidence is particularly lacking for abdominal obesity, which is strongly linked with dementia risk among Asian populations [15]. Degree of abdominal obesity is a direct indicator of central fat distribution, an important driver of metabolic and neurodegenerative diseases [16,17]. China faces a high and steadily increasing prevalence of abdominal obesity [18]. The proportion of abdominally obese middle-aged Chinese adults with a normal body mass index is nearly three times higher than in western populations [19,20]. The Chinese government has introduced nationwide obesity control initiatives since 2016 [21,22]. Such efforts may curb the upward trend of abdominal obesity prevalence, and are likely to reshape the future burden of age-related diseases.

Understanding how trends in abdominal obesity prevalence influence the development and interplay of dementia, FI, and CVD is critical for anticipating healthcare demands in an aging population. This study aims to project the burden of these conditions in China over the next 30 years under three abdominal obesity prevalence trend scenarios: continuation of the current growth trajectory (persistent scenario), stabilization at 2015 prevalence levels (optimal scenario), and a halving of the historical growth trend of abdominal obesity prevalence(improved scenario). Scenario definitions were informed by data from nationally representative aging cohorts and published literature. The impact of abdominal obesity on transitions between disease states and mortality was estimated using the validated IMPACT-China Ageing Model (CAM) [2] multistate Markov model. Results are disaggregated by sex and age group to capture differential disease and mortality burdens between men and women across the life course.

## Method

**Fig 1:**
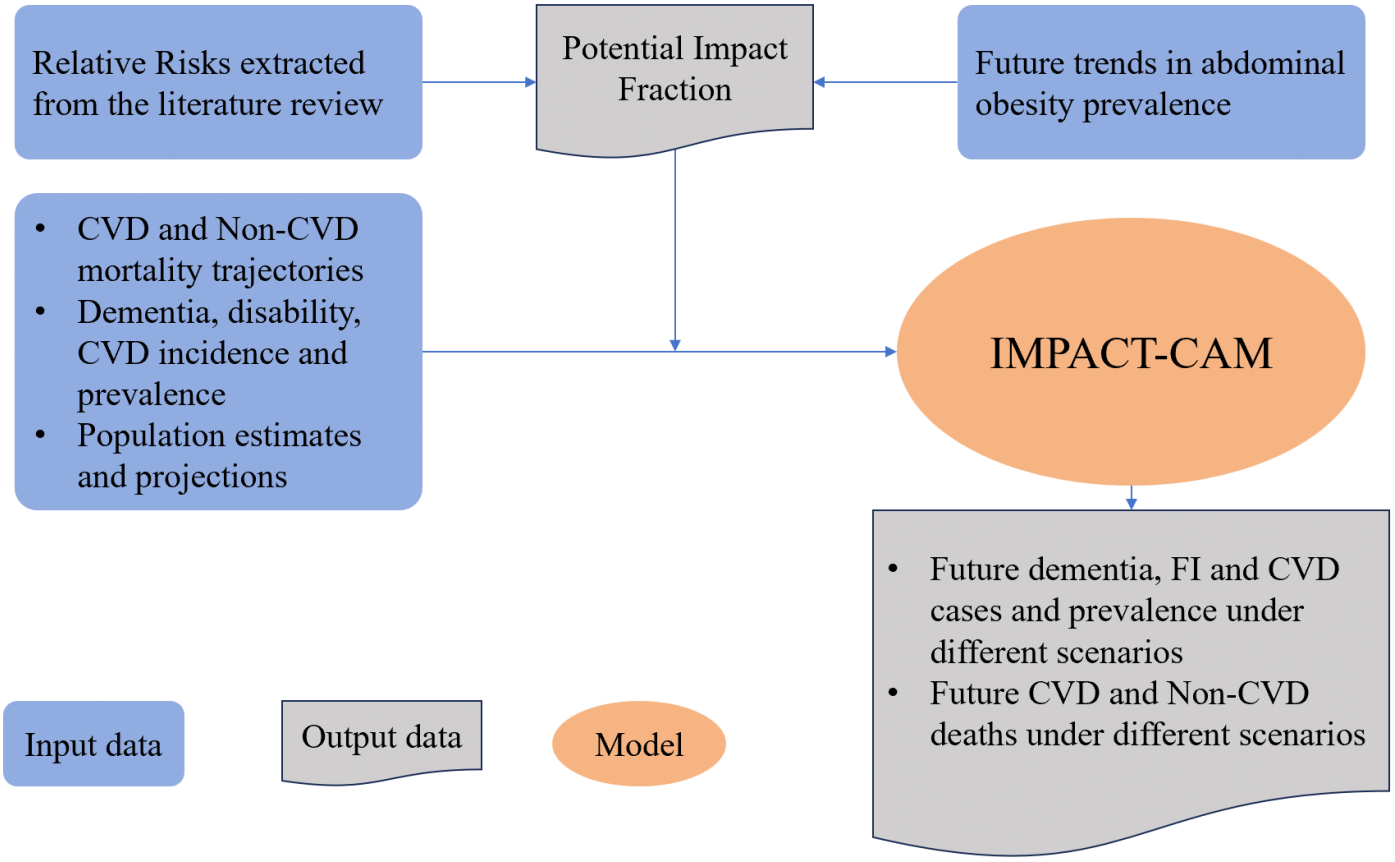
Diagram illustrating steps of the analysis. Notes: CVD: cardiovascular diseases. FI: functional impairment. IMPACT-CAM: IMPACT-China Ageing Model. Relative risks refer to the risks of CVD mortality, non-CVD mortality, dementia incidence, FI incidence, and FI reversal associated with abdominal obesity compared with no abdominal obesity.

Our study proceeded in two main steps. First, we estimated future trends in abdominal obesity prevalence under three plausible scenarios. Second, we quantified the potential impact of these scenarios on dementia, CVD, FI, and mortality in the Chinese older population using our previously validated IMPACT-CAM model [2]. The study flowchart is presented in Fig 1.

### Future trend of abdominal obesity prevalence

We modeled three plausible future scenarios from 2015 to 2050, based on observed trends of abdominal obesity prevalence in the China Health and Nutrition Survey (CHNS, *N* = 17,054 in 2000) [23]. Abdominal obesity was defined as a waist circumference of ≥90 cm for men and ≥85 cm for women, measured at the level of the umbilicus with participants barefoot [24]. Prevalence data from 2000 to 2015 were used for individuals aged 45 years and older, stratified by sex and 5-year age groups. Based on these prevalence trends, we constructed three age- and sex-specific future scenarios (S1 Fig): persistent scenario: a continuation of the annual increase in abdominal obesity observed from 2000 to 2015; optimal scenario: abdominal obesity prevalence remains at 2015 levels, representing the ideal impact of successful intervention; and improved scenario: the rate of annual increase is reduced by 50% compared to the persistent scenario.

### Effect of abdominal obesity prevalence trends on dementia, CVD, FI, and mortality

#### Overview of IMPACT-CAM model.

We used the established IMPACT-CAM model to estimate the effects of alternative abdominal obesity trajectories on future disease burden. IMPACT-CAM is a probabilistic multi-state Markov model that simulates the progression of a healthy Chinese population (aged ≥35 years) from 2015 to 2050 through eight health states defined by the presence or absence of dementia, CVD, FI, their combinations, and two cause-specific mortality states (CVD and non-CVD) (S2 Fig). The model follows the methodological framework of its UK counterpart, IMPACT-BAM [25], and generates age-, sex-, and calendar year–specific estimates and transition probabilities (TPs) using Chinese population–representative aging cohorts and the best available epidemiological data (Section 2 in S1 Appendix, Overview of the IMPACT-CAM model)..

To initialize the model, age- and sex-specific population counts from United Nations statistics were combined with prevalence estimates from the China Health and Retirement Longitudinal Study [26] (CHARLS, 2011–2018; *N* = 25,041) to populate all health states at study baseline (mid-point of the CHARLS survey, 2015). TPs between the eight non-death states (i.e., incidence or recovery rates) were derived from CHARLS, with CVD and FI incidence modeled from age 35 years onwards and dementia incidence from age 50 years. As CHARLS primarily includes participants aged ≥45 years, TPs for ages below 45 were interpolated using the shape-preserving cubic Hermite polynomial method [27]. Detailed TPs between eight non-death states are presented in S3 Fig.

TPs from each health state to CVD and non-CVD death were derived from the Chinese Longitudinal Healthy Longevity Survey [28] (CLHLS, 2002–2005 and 2014–2018; N = 39,747 with recorded causes of death) and were combined with UN and GBD mortality projections to obtain population-level estimates. Additional methodological details are provided in the S1 Appendix (Section 2 in S1 Appendix, Overview of the IMPACT-CAM model), with key model assumptions summarized in S1 Table, and initial numbers of disease prevalence summarized in S2 Table.

Dementia was defined as the co-occurrence of CI and FI, or a self- or informant-reported physician diagnosis of dementia [29]. CI was defined as having impairment in two or more cognitive domains, where impairment in each domain was a factor score at least 1.5 standard deviation (SD) below the education-adjusted mean of adults aged ≥50 years. FI was defined as dependence in one or more of the six basic activities of daily living (BADLs) based on the Katz scale (bathing, dressing, toileting, transferring, continence, and feeding) [30]. CVD was defined as self-reported with a diagnosis of stroke, or CVD (heart attack, heart disease or coronary heart disease).

A more detailed description of the model is available in our previously published paper [2]. Information on the validation of core model estimates against external data sources is provided in the S4 and S5 Figs.

#### Effect of abdominal obesity prevalence trend on transition probabilities.

We assumed that trends in abdominal obesity prevalence under each scenario would modify the relevant IMPACT-CAM TPs, leading to changes in disease burden and mortality. The affected TPs included risk of CVD and non-CVD death, incidence of CVD, dementia, FI, and recovery from FI. To model these changes, we applied an approach based on the potential impact fraction (PIF) for discrete risk factors. PIF accounts for both the prevalence of a risk factor and its associated relative risk (RR), representing the proportional reduction in disease incidence when exposure to the risk factor is modified [31]. The population attributable risk fraction (PARF) is a special case of PIF.


$$\text{PIF}=\frac{\left(P-P^{\prime })\times (RR-\text{1}\right)}{\text{1}+P\times (RR-\text{1})}$$

As defined by the formula above, PIF requires (1) age- and sex-specific abdominal obesity prevalence trends under each scenario over time, which were estimated from CHNS for the persistent scenario (*P*) with scenario-specific adjustments reflecting potential interventions (*P*′) (S1 Fig); and (2) RR comparing exposed with unexposed groups by age, sex and affected TPs. We derived adjusted RRs from published Chinese longitudinal studies, controlling for key sociodemographic and lifestyle factors while avoiding overadjustment for potential intermediates in the causal pathway (e.g., hypertension, diabetes, etc.) [32]. Our review strategy and the extracted RRs with corresponding TPs are provided in S1 Appendix (Section 3 in S1 Appendix, Literature review on relative risks of abdominal obesity on dementia, CVD, and FI) and S3 Table.

For the optimal and improved scenarios, scenario-specific TPs were generated by multiplying the corresponding baseline TP for each age–sex–year stratum by (1 − PIF), yielding PIF-adjusted TPs [33]. A similar approach has been used in IMPACT-BAM scenario analyses [14,34]. Full methodological details are provided in S1 Appendix (Section 4 in S1 Appendix, Adjustment of Transition Probability).

#### Outcomes.

Using above scenario-specific TPs, we recalculated the IMPACT-CAM model to estimate the numbers of dementia, CVD, FI cases, and deaths from 2015 to 2050 under optimal and improved scenarios, and compared them with the results of persistent scenario. Our primary analyses focused on outcomes among adults aged ≥65 years during 2020–2050, with 2015–2020 results used mainly for model validation [2]. We report sex- and age-specific cumulative differences in incident dementia, CVD, FI, and deaths relative to the persistent scenario.

To account for parameter uncertainty, we conducted probabilistic analysis with Monte Carlo simulations. The analysis entailed iterative sampling from specified distributions of the model input parameters to recalculate the outputs. We performed 1,000 iterations to estimate 95% uncertainty intervals (UIs) for the outcome measures. Moreover, we tested the robustness of our results by applying alternative calendar effects to dementia incidence (an annual increase of 2.9% or a decrease of 1.0%) and to CVD incidence (a decline aligned with projected CVD mortality trajectories), and comparing these with the base model that assumed no calendar effects.

## Result

Tables 1 and 2 present the projected disease burden and mortality among individuals aged 65 years and above in China under the persistent scenario. The number of dementia cases is anticipated to rise from 9.8 million (95% UI [9.6, 10.0]) in 2020 to 37.8 million (95% UI [36.7,38.9]) in 2050. Similarly, the number of individuals living with FI and CVD will increase from 25.5 million (95% UI [25.2, 25.7]) to 68.1 million (95% UI [66.7, 69.5]), and from 65.0 million (95% UI [64.8, 65.2]) to 198.3 million (95% UI [196.5, 200.4]) over the same period. The prevalence of these three conditions is projected to rise steeply. The total number of deaths is expected to nearly double, rising from 8.7 million (95% UI [8.7, 8.7]) in 2020 to 17.6 million (95% UI [16.5, 18.7]) in 2050, with CVD accounting for approximately 50% of these deaths. However, the overall mortality rate shows only a modest increase, from 4.9% (95% UI [4.9, 4.9]) in 2020 to 5.6% (95% UI [5.3, 5.9]) in 2050.

**Table 1:** Projected cases and prevalence of dementia, functional impairment, and cardiovascular diseases in Chinese old population aged ≥65 years for the persistent abdominal obesity scenario.

	Dementia cases		FI cases		CVD cases	
	Number (Million)	Prevalence (%)	Number (Million)	Prevalence (%)	Number (Million)	Prevalence (%)
**All**						
2020	9.8 (9.6, 10.0)	5.5 (5.4, 5.6)	25.5 (25.2, 25.7)	14.4 (14.2, 14.5)	65.0 (64.8, 65.2)	36.7 (36.6, 36.9)
2030	19.8 (19.3, 20.3)	8.4 (8.2, 8.6)	40.6 (40.0, 41.2)	17.2 (16.9, 17.4)	117.7 (117.0, 118.4)	49.8 (49.5, 50.0)
2040	31.3 (30.5, 32.2)	10.2 (9.9, 10.5)	58.7 (57.6, 59.7)	19.1 (18.8, 19.4)	178.1 (176.8, 179.5)	58.0 (57.7, 58.3)
2050	37.8 (36.7, 38.9)	12.0 (11.7, 12.3)	68.1 (66.7, 69.5)	21.6 (21.3, 22.0)	198.3 (196.5, 200.4)	63.0 (62.7, 63.3)
**Men**						
2020	3.6 (3.5, 3.7)	4.5 (4.4, 4.6)	9.9 (9.7, 10.0)	12.3 (12.1, 12.4)	26.2 (26.1, 26.4)	32.6 (32.4, 32.8)
2030	7.0 (6.7, 7.2)	6.6 (6.3, 6.8)	15.1 (14.7, 15.4)	14.2 (13.9, 14.5)	46.5 (46.1, 47.0)	43.7 (43.4, 44.1)
2040	11.1 (10.6, 11.5)	8.0 (7.7, 8.3)	21.5 (21.0, 22.1)	15.6 (15.2, 16.0)	70.9 (70.0, 71.8)	51.3 (50.9, 51.7)
2050	13.4 (12.9, 14.0)	9.5 (9.1, 9.9)	25.0 (24.3, 25.8)	17.7 (17.3, 18.1)	79.4 (78.1, 80.7)	56.1 (55.7, 56.6)
**Women**						
2020	6.2 (6.1, 6.3)	6.4 (6.3, 6.6)	15.6 (15.4, 15.8)	16.2 (15.9, 16.4)	38.8 (38.6, 38.9)	40.2 (40.0, 40.3)
2030	12.8 (12.4, 13.3)	9.9 (9.5, 10.2)	25.6 (25.1, 26.1)	19.6 (19.2, 20.0)	71.2 (70.7, 71.7)	54.7 (54.3, 55.0)
2040	20.3 (19.5, 21.0)	12.0 (11.6, 12.4)	37.2 (36.3, 38.0)	22.0 (21.5, 22.5)	107.2 (106.3, 108.1)	63.5 (63.1, 63.9)
2050	24.4 (23.4, 25.3)	14.1 (13.5, 14.6)	43.1 (42.0, 44.2)	24.9 (24.3, 25.5)	118.9 (117.6, 120.4)	68.7 (68.2, 69.1)

Notes: CVD, cardiovascular diseases; FI, functional impairment. The data in parentheses represent the 95% uncertainty intervals. Persistent scenario: The prevalence of abdominal obesity continues to increase.

**Table 2:** Projected numbers and mortality rate of all-cause deaths, cardiovascular disease deaths, and non-cardiovascular disease deaths in Chinese old population aged ≥65 years for the persistent abdominal obesity scenario.

	Total deaths		CVD deaths		Non-CVD deaths	
	Number (Million)	Prevalence (%)	Number (Million)	Prevalence (%)	Number (Million)	Prevalence (%)
**All**						
2020	8.7 (8.7, 8.7)	4.9 (4.9, 4.9)	4.3 (4.3, 4.3)	2.4 (2.4, 2.4)	4.4 (4.4, 4.4)	2.5 (2.5, 2.5)
2030	12.2 (11.7, 12.6)	5.1 (4.9, 5.3)	5.7 (5.5, 6.0)	2.4 (2.3, 2.5)	6.4 (6.0, 6.8)	2.7 (2.5, 2.9)
2040	15.8 (15.0, 16.6)	5.2 (4.9, 5.4)	7.3 (6.9, 7.8)	2.4 (2.2, 2.5)	8.5 (7.8, 9.2)	2.8 (2.5, 3.0)
2050	17.6 (16.5, 18.7)	5.6 (5.3, 5.9)	8.3 (7.7, 8.9)	2.6 (2.4, 2.8)	9.3 (8.4, 10.2)	3.0 (2.7, 3.2)
**Men**						
2020	4.5 (4.5, 4.5)	5.6 (5.6, 5.7)	2.1 (2.1, 2.1)	2.6 (2.6, 2.6)	2.5 (2.5, 2.5)	3.1 (3.1, 3.1)
2030	6.1 (5.8, 6.5)	5.8 (5.4, 6.1)	2.7 (2.5, 2.9)	2.5 (2.3, 2.7)	3.5 (3.2, 3.8)	3.2 (3.0, 3.5)
2040	7.8 (7.2, 8.5)	5.7 (5.2, 6.1)	3.4 (3.1, 3.7)	2.5 (2.3, 2.7)	4.4 (3.9, 4.9)	3.2 (2.8, 3.6)
2050	8.7 (7.9, 9.5)	6.1 (5.6, 6.7)	4.0 (3.5, 4.4)	2.8 (2.5, 3.1)	4.7 (4.1, 5.4)	3.4 (2.9, 3.8)
**Women**						
2020	4.1 (4.1, 4.1)	4.3 (4.3, 4.3)	2.2 (2.2, 2.2)	2.3 (2.3, 2.3)	1.9 (1.9, 1.9)	2.0 (2.0, 2.0)
2030	6.0 (5.7, 6.3)	4.6 (4.4, 4.8)	3.1 (2.9, 3.2)	2.4 (2.2, 2.5)	3.0 (2.7, 3.2)	2.3 (2.1, 2.4)
2040	8.0 (7.4, 8.5)	4.7 (4.4, 5.1)	3.9 (3.5, 4.2)	2.3 (2.1, 2.5)	4.1 (3.6, 4.6)	2.4 (2.2, 2.7)
2050	8.9 (8.2, 9.7)	5.2 (4.7, 5.6)	4.3 (3.9, 4.8)	2.5 (2.3, 2.8)	4.6 (4.0, 5.2)	2.7 (2.3, 3.0)

Notes: CVD, cardiovascular diseases. The data in parentheses represent the 95% uncertainty intervals. Persistent scenario: The prevalence of abdominal obesity continues to increase.

**Fig 2:**
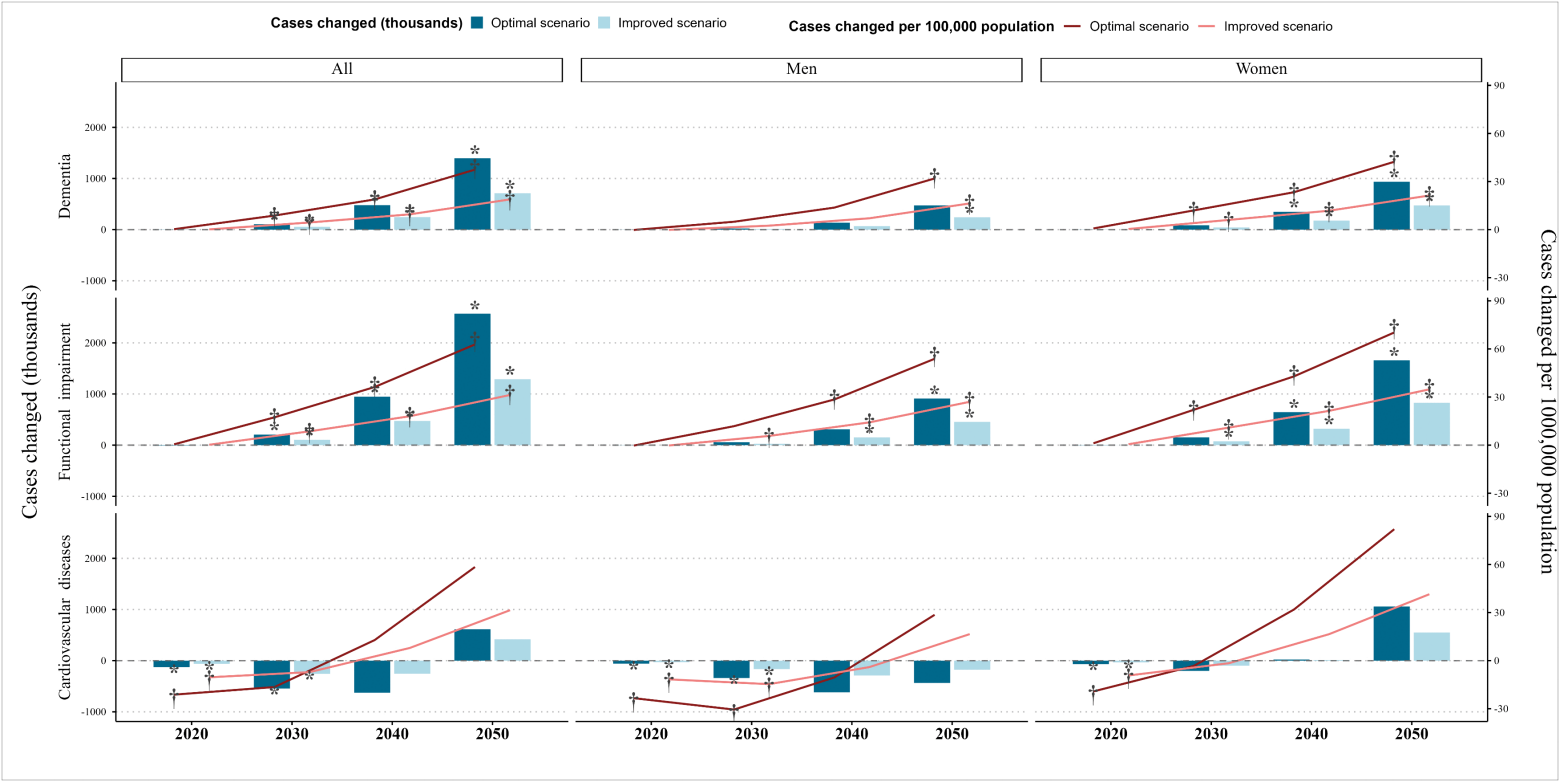
Number of incident cases of dementia, functional impairment and cardiovascular diseases changed (cumulative since 2015) for optimal and improved scenario vs. persistent scenario in Chinese population aged ≥65 years. Notes: CVD, cardiovascular diseases; FI, functional impairment. Persistent scenario: prevalence of abdominal obesity continues to increase. Optimal scenario: prevalence of abdominal obesity remains unchanged. Improved scenario: growth rate of abdominal obesity prevalence is reduced by 50%. *and † represent that the estimation is statistically significant (95% uncertainty interval excludes 0). All values represent cumulative amounts since 2015. The corresponding numerical estimates and 95% uncertainty intervals are presented in detail in S4 Table.

The relative changes in disease burden compared to the other two abdominal obesity scenarios are illustrated in Fig 2 (Actual numbers are provided in S4 Table). In the optimal scenario, there is a slight increase in the number of dementia and FI cases by 2050 compared to the persistent scenario, with cumulative increases of 1396.0 thousand (95% UI [589.1, 2293.9]) and 2570.6 thousand (95% UI [1081.0, 4024.9]), respectively. The improved scenario showed a similar pattern to the optimal scenario, with smaller increases of 711.4 thousand (95% UI [294.4, 1150.3]) dementia cases and 1289.5 thousand (95% UI [569.5, 2034.2]) FI cases, approximately half of those estimated under the optimal scenario. For CVD, a decline in the number of cases is projected by 2030, although longer-term changes (2040–2050) remain uncertain. By 2050, the increase in dementia and FI among men is projected to be lower than that among women. and the decline in CVD prevalence is more pronounced among men. Age-stratified results (S6 and S7 Figs) show that: disease burden decreases in younger-old adults (aged 65–69 years) but increases substantially in the oldest-old populations (≥80 years).

**Fig 3:**
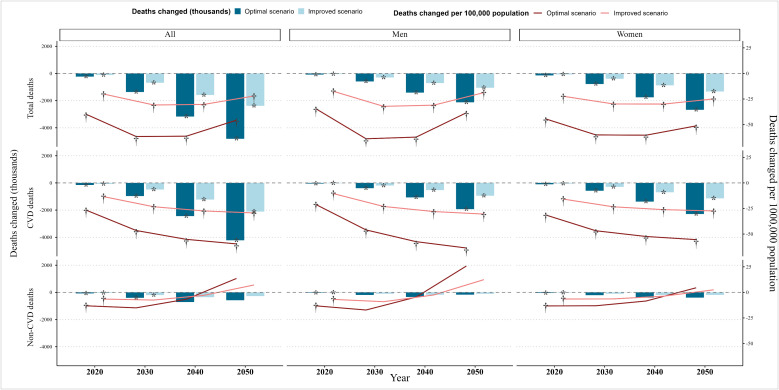
Number of deaths changed (cumulative since 2015) for optimal and improved scenario vs. persistent scenario in Chinese population aged ≥65 years. Notes: CVD, cardiovascular diseases. Persistent scenario: prevalence of abdominal obesity continues to increase. Optimal scenario: prevalence of abdominal obesity remains unchanged. Improved scenario: growth rate of abdominal obesity prevalence is reduced by 50%. *and † represent that the estimation is statistically significant (95% uncertainty interval excludes 0). All values represent cumulative amounts since 2015. The corresponding numerical estimates and 95% uncertainty intervals are presented in detail in S5 Table.

Fig 3 shows the changes in mortality burden in individuals aged 65 and above under two alternative abdominal obesity scenarios (Actual numbers are provided in S5 Table). In the optimal scenario, compared to the persistent scenario, a cumulative reduction in the number of deaths by 2020 is projected to be 229.3 thousand (a decrease of 39.8/100,000). By 2050, the cumulative reduction in deaths is expected to reach 4807.8 thousand (a decrease of 45.7/ 100,000), with the majority (>80%) of avoided deaths attributed to reductions in CVD mortality. The reduction in deaths is more pronounced in women than in men. In the improved scenario, these changes mirror the constant scenario but with approximately 50% magnitude of change. These patterns remain robust, as alternative calendar effects on disease incidence did not significantly alter the overall directions of change between scenarios (S8 and S9 Figs).

## Discussion

Using the well-validated multistate IMPACT-CAM that accounts for competing risks of comorbidities and mortality, we projected the long-term impact of abdominal obesity control on dementia, CVD and FI in China. Our findings suggest that while abdominal obesity interventions can effectively reduce CVD incidence and mortality, they may also shift the disease burden among older Chinese adults toward dementia and FI. This paradox arises from the demographic consequence of reduced premature mortality, particularly from CVD, which increases the number of individuals surviving into advanced age, where the risk of cognitive and functional decline is highest. This effect is more pronounced among women.

Unlike prior modeling studies that focused on single-disease outcomes, our study employed a multistate Markov model to jointly estimate transitions across dementia, CVD, FI, and mortality. Two earlier studies projected future dementia burden based on changes in obesity prevalence using population-based macrosimulation models that did not account for comorbidity interactions or competing mortality [11,12]. Another study considered life expectancy but remained limited to cardiometabolic endpoints, without modeling how reduced obesity-related mortality might alter the future distribution of age-related diseases [13]. By integrating multiple disease pathways and competing risks into a unified model, our approach provides a more realistic estimation of how interventions may shift, rather than simply reduce, the overall burden of chronic disease in aging populations.

Consistent with this, our model estimated a maximum mortality reduction of 27.3% attributable to abdominal obesity control, aligning with known epidemiological evidence [35]. For dementia and FI, the protective effects of obesity control appear to be offset by a survival-driven accumulation of older adults at risk. This dynamic explains the net increase in dementia-related FI observed in our simulations. A similar paradox has been reported in UK modeling of hypertension control, where reductions in blood pressure were associated with 24,500 additional dementia cases by 2060 due to increased survival [14]. The increase in dementia and FI cases was most pronounced among those aged 80 years and older, outweighing the reductions seen in individuals under 70. These findings underscore that population aging, rather than modifiable risk factors alone, will remain the primary driver of future dementia and FI burdens [2,36].

These shifts in disease burden are marked by sex differences, a dimension largely overlooked in prior modeling. We found that abdominal obesity interventions led to a somewhat greater increase in dementia and FI cases among women than men, alongside a smaller reduction in CVD. Women in our study sample had a higher baseline prevalence of abdominal obesity (S1 Fig) and slightly lower associated RRs for CVD and FI (S2 Table) than men. As implied by the PIF function, these differences yield smaller proportional changes in adjusted TPs for women, and therefore smaller benefits from reducing abdominal obesity. These differences might reflect sex-specific biological mechanisms. A 10-year Japanese cohort study reported that visceral fat area was associated with cognitive decline in men but not in women, which the authors suggested may indicate a compensatory role of visceral fat in postmenopausal women with low estrogen levels [37]. Estrogen supports hippocampal neuroplasticity and regulates Aβ production [38] and higher estrogen levels have been linked to reduced risks of cognitive decline, Alzheimer’s disease, and CVD in older women [39–41]. Reductions in abdominal obesity may therefore attenuate this hormonal protection, contributing to the relatively larger projected disease burden in women. Furthermore, our projections show that women experience larger declines in mortality than men (Fig 3). Greater mortality reductions expand the number of women surviving to very old ages, when dementia and CVD risks are highest. In contrast, the smaller mortality decline among men likely produces a more selective survivor cohort with lower underlying disease risks [42]. Together, these findings highlight the need to incorporate sex-specific risk profiles, biological pathways, and mortality trajectories when modeling population-level impacts of obesity interventions in aging societies.

The public health implications of our findings are significant. As China implements national strategies to curb obesity [21,22], it is crucial to anticipate how these efforts will reshape the disease burden in an aging population. While obesity control is essential for reducing premature CVD mortality, our results highlight the unintended consequence of increased dementia and FI among the oldest-old. This calls for integrated public health strategies that extend beyond single-disease prevention to address the complex interactions among chronic conditions across the life course. Interventions should also be tailored by sex, with targeted strategies for risk factor management, cognitive screening, and long-term care planning. As population aging accelerates, cross-sectoral coordination will be essential to ensure that gains in life expectancy translate into longer, healthier lives.

A major strength of our study lies in the structure of the IMPACT-CAM model, which enables simulation of complex, interrelated disease pathways using nationally representative data from China. Limitations should also be noted. We used the PIF approach to translate trends in abdominal obesity prevalence into changes in the risks of dementia, related comorbidities, and mortality. This approach rests on the assumption that other risk factors do not need to be accounted for [43]. We used RRs adjusted only for basic sociodemographic and lifestyle factors so the accuracy of projections within each scenario is depends on the extent to which abdominal obesity indexes all the important risk factors. Our study focuses on the relative differences across obesity-prevalence scenarios, reflecting alternative prevention strategies, and comparative inferences are therefore reasonable. Given the broad range of modifiable dementia risk factors [8], future modeling incorporating multiple co-occurring risk factors is warranted to assess the impact of multidimensional intervention strategies on dementia and its comorbidities.

In summary, our study suggests that controlling abdominal obesity may shift China’s chronic disease burden in the aging population from CVD toward dementia and FI. While such interventions are effective in reducing mortality and CVD incidence, they may have limited impact on age-related cognitive and physical decline, posing new challenges for healthy aging. These findings call for integrated, sex-sensitive, and multi-risk-factor public health strategies to optimize both longevity and quality of life in aging societies.

## Supporting information

S1 Appendix Supplementary material. Overview of the IMPACT-CAM model, different abdominal obesity scenarios, the TP adjustment mechanism, age-stratified results, and sensitivity analysis results. Notes: IMPACT-CAM, IMPACT-Chinese Ageing Model; TP, transition probability.
(DOCX)

S1 Fig Projected prevalence trajectories in abdominal obesity in Chinese population by age and sex under the three scenarios. (TIF)

S2 Fig IMPACT-CAM structure. Notes: IMPACT-CAM framework was developed in reference to the IMPACT-BAM. Transition to death states 9 and 10 are possible from any state in the model, as noted by TP_*i*,9_ and TP_*i*,10_ (*i* = 1–8). The structure is a simplified representation of the model, the figure shows only the major paths. CVD, cardiovascular diseases; FI, functional impairment; CI, Cognitive Impairment; CIND, Cognitive impairment no dementia; Dis-free, free of CVD, CI, FI or Dementia; IMPACT-CAM, IMPACT-China Ageing Model.
(TIF)

S3 Fig Plot of initial transition probabilities. Notes: TP, transition probability.
(TIF)

S4 Fig Dementia Cases Comparison between IMPACT-CAM (2018) and COAST Study in 2018. Notes: IMPACT-CAM, IMPACT-Chinese Ageing Model; COAST, Cohort of Ageing Study; Ref: COAST Study.
(TIF)

S5 Fig Comparison of the Number of Cardiovascular (CVD) and Non-cardiovascular (Non-CVD) Death Cases Estimated by IMPACT-CAM and GBD (2019) from 2015 to 2019. Notes: IMPACT-CAM, IMPACT-Chinese Ageing Model; GBD, Global Burden of Diseases. Ref: GBD 2019.
(TIF)

S6 Fig Number of incident cases of dementia, functional impairment and cardiovascular diseases changed (cumulative since 2015) for optimal and improved scenario versus persistent scenario in Chinese population aged ≥65 years (age-stratified). Notes: Combined estimate for both sexes. CVD, cardiovascular diseases; FI, functional impairment. Persistent scenario: prevalence of abdominal obesity continues to increase; Optimal scenario, prevalence of abdominal obesity remains unchanged. Improved scenario: growth rate of abdominal obesity prevalence is reduced by 50%. * represent that the estimation is statistically significant (95% uncertainty interval excluded 0). All values represent cumulative amounts since 2015.
(TIF)

S7 Fig Number of incident cases of dementia, functional impairment and cardiovascular changed per 100,000 population (cumulative since 2015) for optimal and improved scenario versus persistent scenario in Chinese population aged ≥65 years (age-stratified). Notes: Combined estimate for both sexes. CVD, cardiovascular diseases; FI, functional impairment. Persistent scenario: prevalence of abdominal obesity continues to increase. Optimal scenario: prevalence of abdominal obesity remains unchanged. Improved scenario: growth rate of abdominal obesity prevalence is reduced by 50%. * represent that the estimation is statistically significant (95% uncertainty interval excluded 0). All values represent cumulative amounts since 2015.
(TIF)

S8 Fig Number of incident cases of dementia, functional impairment and cardiovascular changed (Thousands, cumulative since 2015) for optimal and improved scenario versus persistent scenario in Chinese population aged ≥65 years (different calendar effects of dementia incidence, CVD incidence). Notes: CVD, cardiovascular diseases; FI, functional impairment.
(TIF)

S9 Fig Number of incident cases of dementia, functional impairment and cardiovascular changed per 100,000 population (cumulative since 2015) for optimal and improved scenario versus persistent scenario in Chinese population aged ≥65 years (different calendar effects of dementia incidence, CVD incidence). Notes: CVD, cardiovascular diseases; FI, functional impairment.
(TIF)

S1 Table Summary of assumptions underlying the IMPACT-CAM model. Note: IMPACT-CAM, IMPACT-Chinese Ageing Model.
(DOCX)

S2 Table Input Baseline Prevalence (%) at 2015 of IMPACT-CAM. Notes: CVD, cardiovascular diseases; FI, functional impairment; CI, Cognitive Impairment; CIND, Cognitive impairment no dementia; Dis-free, free of CVD, CI, FI or Dementia.
(DOCX)

S3 Table Transition probabilities affected by the change in abdominal obesity. Notes: CVD, cardiovascular diseases; TP, transition probability; RR, relative risk. RR represents the relative risks of each outcome associated with abdominal obesity compared with no abdominal obesity. Estimated from Chinese longitudinal studies, adjusted for key sociodemographic and lifestyle factors but not for potential intermediates in the causal pathway (e.g., hypertension, diabetes).
(DOCX)

S4 Table Number of incident cases of dementia, FI and cardiovascular disease avoided (cumulative since 2015) for optimal and improved scenario versus persistent scenario in Chinese population aged ≥65 years. Notes: CVD, cardiovascular diseases; FI, functional impairment. The data in parentheses represent the 95% uncertainty intervals. Persistent scenario: The prevalence of abdominal obesity continues to increase. Optimal scenario: prevalence of abdominal obesity remains unchanged. Improved scenario: growth rate of abdominal obesity prevalence is reduced by 50%.
(DOCX)

S5 Table Number of deaths avoided (cumulative since 2015) for optimal and improved scenario versus persistent scenario in Chinese population aged ≥65 years. Notes: CVD, cardiovascular diseases; FI, functional impairment. The data in parentheses represent the 95% uncertainty intervals. Persistent scenario: The prevalence of abdominal obesity continues to increase. Optimal scenario: prevalence of abdominal obesity remains unchanged. Improved scenario: growth rate of abdominal obesity prevalence is reduced by 50%.
(DOCX)

## Abbreviations

BADLs: basic activities of daily living
IMPACT-CAM: IMPACT-China Ageing Model
CHARLS: China Health and Retirement Longitudinal Study
CI: cognitive impairment
CVD: cardiovascular disease
FI: functional impairment
PARF: population attributable risk fraction
PIF: potential impact fraction
RR: relative risk
SD: standard deviation
TPs: transition probabilities
UI: uncertainty interval
