# Supplementary material for: Impact of abdominal obesity prevalence trends on dementia, cardiovascular disease, functional impairment, and mortality in older Chinese adults: A Markov scenario simulation, 2020–2050
Source: PLoS Med. 2026 Apr 7;23(4):e1004970. doi: 10.1371/journal.pmed.1004970 (PMC13082697; doi:10.1371/journal.pmed.1004970)
Supplement: S1 Appendix — Overview of the IMPACT-CAM model, different abdominal obesity scenarios, the TP adjustment mechanism, age-stratified results, and sensitivity analysis results. Notes: IMPACT-CAM, IMPACT-Chinese Ageing Model; TP, transition probability. (DOCX) [file pmed.1004970.s001.docx]

**Impact of Abdominal Obesity Prevalence Trends on Dementia, Cardiovascular Disease, Functional impairment, and Mortality in Older Chinese Adults: A Markov Scenario Simulation, 2020–2050**

**S1 Appendix**

**Content:**

[1 Future trends in abdominal obesity prevalence 3](#_Toc222954529)

[2 Overview of IMPACT-CAM model 4](#_Toc222954530)

[2.1 Data Sources Used in IMPACT-China Ageing Model (CAM) 4](#_Toc222954531)

[2.2 Overview of IMPACT-CAM 5](#_Toc222954532)

[2.3 Case and State Definitions 5](#_Toc222954533)

[2.4 Prevalence Calculations 6](#_Toc222954534)

[2.5 Method of Transition Probability 6](#_Toc222954535)

[2. 6 Uncertainty Interval of the Estimates 9](#_Toc222954536)

[3 Literature review on relative risks of abdominal obesity on dementia, CVD and functional impairment 10](#_Toc222954537)

[4 Adjustment of Transition Probability 11](#_Toc222954538)

[5 Age-stratified results 12](#_Toc222954539)

[6 Sensitivity analysis for different calendar effect 13](#_Toc222954540)

[References 14](#_Toc222954541)

1 Future trends in abdominal obesity prevalence

Data on physical examinations from respondents of the China Health and Nutrition Survey (CHNS)(1)2000–2015 were used (data access: https://dataverse.unc.edu/dataverse/chns ). To model prevalence trends, we fitted sex- and age-specific trajectories of abdominal obesity with an inverse proportional function. Given that the prevalence of abdominal obesity in China is projected to continue increasing, we defined three scenarios: persistent (continued growth in prevalence), optimal (no further growth), and improved (a slower growth rate)

.

2 Overview of IMPACT-CAM model

2.1 Data Sources Used in IMPACT-China Ageing Model (CAM)

**China Health Aging and Retirement Longitudinal Study** (2)**:** China Health Aging and Retirement Longitudinal Study (CHARLS) is a nationwide longitudinal survey that focuses on middle-aged and elderly individuals (aged 45 years and above) predominantly residing in the community. The study encompasses 28 provinces, autonomous regions, and municipalities across China. Employing a multi-stage and random probability sampling method, the initial wave of the study conducted in 2011 involved over 17,000 participants. Subsequent waves were conducted at intervals of two to three years, with the most recent survey taking place in 2018 (achieving a follow-up rate of over 88% in each subsequent wave). Comprehensive information regarding the study's design and data quality can be found in the cohort profile. (Accessed at: https://charls.pku.edu.cn/en/)

**Chinese Longitudinal Healthy Longevity Study, (CLHLS)** (3)**:** The Chinese Longitudinal Healthy Longevity Survey (CLHLS) is a nationwide longitudinal survey that focuses on elderly individuals aged 65 years and above. The survey design includes a multistage oversampling approach from 22 provinces, specifically targeting older adults. CLHLS comprises a total of eight surveys conducted between 1998 and 2018. The surveys conducted in 1998 and 2000 specifically focused on individuals aged 80 years and above, while from 2002 onwards, the age range of survey respondents was expanded to include individuals aged 65 years and above. In order to comprehensively assess the mortality among individuals in China, data from 2002 onwards were selected for the CLHLS sample in this study. (Access at: https://opendata.pku.edu.cn/dataverse/CHADS)

**United Nations World Population Prospects 2022 (UN 2022)** (4)**:** The 2022 Revision of World Population Prospects serves as the twenty-seventh official edition of population estimates and projections by the Population Division of the Department of Economic and Social Affairs of the United Nations Secretariat. It encompasses population projections until the year 2100, offering a range of plausible scenarios at the global, regional, and national levels. For China, the projection for total fertility begins at a lower fertility level compared to the 2019 revision. Recent estimates of total fertility have been revised downwards, considering the findings of the 2020 census and the availability of new health statistics. These revisions are based on registered births and data from the latest census and surveys conducted by the National Bureau of Statistics up to 2021. (Access at: https://population.un.org/wpp/)

**Global Burden of Diseases (GBD)** (5): The Global Burden of Disease (GBD) study serves as a comprehensive tool that offers a holistic perspective on mortality and disability, encompassing various dimensions such as countries, time, age, and sex. Its objective is to quantify the health burden attributed to numerous diseases, injuries, and risk factors, with the aim of facilitating healthcare system improvements and reducing health disparities. In the GBD 2019 study, China's estimation was conducted at the subnational level. The Cause of Death database for China draws upon two primary sources of data: surveillance data from the China Disease Surveillance Points (DSP) system and vital registration (VR) data collected by the Chinese Centre for Disease Control and Prevention (CDC) . (Access at: <https://vizhub.healthdata.org/gbd-results/>)

2.2 Overview of IMPACT-CAM

IMPACT-CAM is a population-based discrete-time probabilistic Markov model that follows the progression of a healthy Chinese population (aged 35+ years) across ten different health states characterised by the presence or absence of cardiovascular disease (CVD), cognitive impairment (CI), and functional impairment (FI) to death from CVD and non-CVD causes to 2050. The model structure developed in accordance with IMPACT-BAM (a UK-based model) ^(6, 7)^.

$State_{1}$ represents a health state where individuals are free from CVD, CI and FI. Building upon this, $State_{2},State_{4}, State_{8}$ correspond to CVD only, CI only (cognitive impairment without dementia), and FI only (functional impairment not related to CVD or dementia), respectively; while $State_{3} State_{5}, State_{6}, State_{7}$are the comorbidities of CVD and CI, CVD and FI, CVD and dementia (CI and FI).$State_{9}$ and $State_{10}$ denote death from CVD causes and death from non-CVD causes, respectively.

Prior to simulation, we populated each state in the model based on UN population estimates in 2015 (start year) and prevalence of the above conditions from CHARLS, except for the new cohort of 35-year-olds that entered the system through the disease-free state. The simulation allows individuals to move to other states in the model, governed by one-year transition probabilities by age, sex, and calendar year. Possible transitions between states are indicated by arrows in **S2 Fig**.

2.3 Case and State Definitions

**Cognitive Impairment** $\boldsymbol{(}\boldsymbol{Stat}\boldsymbol{e}_{\mathbf{3}}\mathbf{,}\boldsymbol{Stat}\boldsymbol{e}_{\mathbf{4}}\boldsymbol{)}$**:** Cognitive impairment (CI) was detected by the factor scores generated by confirmatory factor analysis (8). Three-factor cognitive structures including orientation, memory and executive and language functions were identified in both cohorts (8), and factor scores were used to calibrate cognitive scores between two cohorts. CI is defined as impairment in two or more domains of cognitive function. Common latent cognitive domains of CHARLS and CLHLS. Impairment in each cognitive domain was defined as a factor score of 1∙5 standard deviations or lower below the mean of the population aged 50+ years with the same level of education (9). To account for transient of CI, participants were considered to be free of CI if their cognitive function score improved by more than 1 standard deviation at the next survey in two or more cognitive domains (10).

**Functional impairment** $\boldsymbol{(Stat}\boldsymbol{e}_{\mathbf{5}}\mathbf{,}\boldsymbol{Stat}\boldsymbol{e}_{\mathbf{6}}\boldsymbol{,Stat}\boldsymbol{e}_{\mathbf{7}}\mathbf{,}\boldsymbol{Stat}\boldsymbol{e}_{\mathbf{8}}\boldsymbol{)}$**:** Functional impairment (FI) is defined as independently performing one or more basic activities of daily living based on the Katz-scale (11), including (1) bathing, (2) dressing, (3) going to the toilet, (4) transfer, (5) continence, and (6) feeding both in CHARLS and CLHLS. Detailed coding was shown in previous literature (8). In CLHLS, the answers for activities of daily living (BADLs) are "1. Without assistance from people; 2. Need assistance partly; 3. Need assistance completely". Option 1 was considered as without functional impairment (12). In CHARLS, the answer for ADLs are “1. No, I don’t have any difficulty; 2. I have difficulty but can still do it; 3. Yes, I have difficulty and need help; 4. I cannot do it” (13). We used the following criteria to define functional impairment: (1) participants who were dependent on one or more ADLs with functional impairment. (2) participants who were independent in all six ADLs were defined as without FI. (3) Additionally, For participants who missed some items but remained independent in either bathing or all IADLs, they were classified as no FI, as bathing and IADLs are relatively more complex than other activities. Our definition of FI thus captures numbers of people with difficulty maintaining basic self-care independence and require supportive care on a daily basis. If participants reported FI once (excluding those in the last wave) but fully recovered to perform BADL independently in all subsequent waves, they were considered to be transient and not categorized as having FI.

**Dementia** $\boldsymbol{(Stat}\boldsymbol{e}_{\mathbf{6}}\mathbf{,}\boldsymbol{Stat}\boldsymbol{e}_{\mathbf{7}}\boldsymbol{)}$**:** Participants with dementia were defined as both CI and FI or self-reported physician-diagnosed dementia. Informant reports of doctor diagnosis of dementia help identify cases when participants were unable to participate in the study (i.e. 32% without cognitive scores). CHARLS does not directly ask about dementia but rather focuses on self-reported memory-related disorders (such as Parkinson's, dementia, etc.), this study determined the proportion of dementia cases among those reporting memory-related disorders by referencing the ratio of Parkinson's and dementia prevalence derived from GBD 2019. This algorithmic case definition conforms to DSM-5 and ICD-10 criteria for dementia, and has previously been validated against a nationwide population-based survey.^7^

**Cardiovascular disease** $\boldsymbol{(Stat}\boldsymbol{e}_{\mathbf{2}}\mathbf{,}\boldsymbol{Stat}\boldsymbol{e}_{\mathbf{3}}\boldsymbol{,Stat}\boldsymbol{e}_{\mathbf{5}}\mathbf{,}\boldsymbol{Stat}\boldsymbol{e}_{\mathbf{6}}\boldsymbol{)}$**:** Cardiovascular disease (CVD) was defined as self-reported with a diagnosis of cardiovascular disease (heart attack, heart disease or coronary heart disease), or stroke in CHARLS and CLHLS.

**CVD and non-CVD death**$\boldsymbol{(}\boldsymbol{Stat}\boldsymbol{e}_{\mathbf{9}}\mathbf{,}\boldsymbol{Stat}\boldsymbol{e}_{\boldsymbol{10}}\boldsymbol{)}$**:** CVD and non-CVD death are defined by the cause of death of the decedent participants. Cause of death was obtained from interviews with the decedents’ relatives. We include CVD and non-CVD death states, as CVD is the leading cause of mortality in China (14), and fundamentally influences dementia prevalence. Competing risks due to death from non-CVD causes such as cancer and COPD are accounted for via an aggregated non-CVD death state.

2.4 Prevalence Calculations

CHARLS provides a nationally representative sample of middle-aged and older Chinese adults, covering a wider geographic and age range compared to CLHLS. Thus, data from CHARLS 2011-2018 was utilized to estimate the prevalence of cardiovascular disease (CVD), cognitive impairment (CI), dementia, and functional impairment (FI) for the model baseline.

To improve statistical power, four waves of CHARLS data were pooled to estimate prevalence of health status of the mid-point of the cohort, which is 2015 (projection baseline). Logistic regression models were used to estimate the prevalence of each state (CVD, CI, dementia, and FI). Additionally, for CI and dementia prevalence calculations, age squared was incorporated to indicate the quadratic effect of age with CI or dementia (6, 15, 16).

$Logit(CVD or FI)= age_{35}+sex+age_{35}*sex$ (1)

$Logit(CI or Demenetia)= age_{50}+sex+age_{50}*sex+ {age}_{50}^{2}$ (2)

Where $age_{50}$ and $age_{35}$ were individual age centred at 50 and 35 years old. Given the limited number of cases, it is assumed that the prevalence among individuals under the age of 50 equals to zero. It is also assumed that the prevalence of cardiovascular disease and dementia are independent of each other, and then calculated the prevalence of comorbidity states. The prevalence estimated from CHARLS was also compared to external data in CVD (17), FI (18), and dementia (19),demonstrating representation of the Chinese population.

2.5 Method of Transition Probability

**2.5.1 Transition probabilities between** **health states (i =1-8)**

CHARLS 2011-2018 was used to estimate transition probabilities (TPs) (i.e., incidence or recovery rates) between CVD, CI and FI from the model baseline. TPs were estimated as functions of age and sex using incident cases occurring between wave n and wave n+1 in CHARLS, based on pooled data referenced to the survey midpoint (2015). To account for potential non-linear relationships between TPs and age, a quadratic age term was included, considerations of data distribution and model fit. The equations for calculating the 2-year TPs of CVD, CI, FI and FI recovery were shown in the formula below:

For incidence of CVD,

$Logit\left( CVD incidence \right)= age_{35}+sex+age_{35}*sex+state_{j}$ (3)

$P(CVD)$ when $state_{j}$ is the non-CVD states (1, 4 and 8) and transitions to CVD states (states 2, 3 and 5), where $age_{35}$ were individual age centred at 35 years old.

For incidence of CI,

$Logit\left( CI incidence \right)= age_{50}+sex+age_{50}*sex+ {age}_{50}^{2}+state_{i}$ (4)

$P(CI)$ is for age- and sex-specific TPs from $state_{i}$, a non-CI state (i=1, 2 and 8) to CI states (states 4, 3 and 7); an additional quadratic term of age was added to indicate CI incidence grows exponentially with age. The transitions were assumed to start from 50 years onwards.

In IMPACT-CAM, we defined CVD-only (states 2) and CI-only (states 4) as mutually exclusive (i.e. a patient who is in the CVD-only state does not have CI at the same time and vice versa), thus $p_{1,3}$ equal to $P\left( CVD\cap CI \right)$,

$p_{1,3}$ = $P(CVD)*P(CI)$ (5)

Similarly, for $p_{8,6}$.

To calculate the transition probability $p_{1,4} and p_{1,2}$, we subtract the proportion of patients who have both CVD and CIND ($p_{1,3})$, as

$p_{1,2}=P\left( CVD \right)-p_{1,3}$ (6)

$p_{1,4}=P\left( CIND \right)-p_{1,3}$ (7)

For incidence of FI,

$Logit\left( FI incidence \right)=age_{35}+sex+age_{35}*sex+state_{m}$ (8)

$P(FI)$ when $state_{m}$ is the non-FI states (1, 2, 3 and 4) and transitions to FI states (states 5, 6, 7 and 8), where $age_{35}$ were individual age centred at 35 years old.

And recovery from FI,

$Logit\left( FI recovery \right)=age_{35}+sex+age_{35}*sex+state_{n}$ (9)

$P(FI recovery)$ when $state_{n}$ is the FI states (5, 6, 7 and 8) and transitions to FI states (states 1, 2, 3 and 4), where $age_{35}$ were individual age centred at 35 years old.

The recurrent state transition probabilities $p_{i,i}$ were calculated using the following formula, while $i$ is from 1 to 8.

$p_{i,i}=1- \sum_{j=1}^{J} p_{i,j}$ (10)

$j$ is a vector containing the states (other than $i$ itself) to where a transition occurs.

For all TPs calculated, 1-year incidence was calculated by ${TP}_{1year}=1- e^{\left( \frac{\ln\left( 1-{TP}_{2 years} \right)}{2} \right)},$where ${TP}_{2 years}$ was the 2-year incidence and ${TP}_{1}$ was the 1-year incidence (20). TPs or incidence of CVD, FI and CI by age and sex were consistent with age- and sex-specific incidence values obtained from CHARLS in the mid-point time close to the whole China based on the multi-stage random sampling.

**2.5.2 Transition probabilities from health states to death states (TPi,9 and TP i,10, i=1-8)**

For the calculation of mortality for all eight health states (${State}_{1-8}$) transitioning to CVD or non-CVD death (${State}_{9,10}$), we employed a three-step process. Population-level projections of CVD and non-CVD mortality were derived from UN and GBD mortality data using a Bayesian Age–Period–Cohort (BAPC) model; state-specific and baseline sex-specific hazard ratios (HRs) for CVD and non-CVD death were estimated from CLHLS; and these health state– and sex-specific ratios were then applied to population mortality rates to obtain age-, sex-, and year-specific TPs.

**Step 1: Estimating population-level CVD and non-CVD mortality.**

We first obtained age-specific all-cause mortality probabilities through 2050 from the UN 2022 projections. Because the UN data do not include CVD- and non-CVD-specific mortality, we integrated GBD cause-specific mortality rates for China (1990–2020) with UN mortality projections (1990–2050, constant mortality scenario) using a BAPC model to estimate future CVD and non-CVD mortality. The Bayesian framework yields posterior distributions for cause-specific death probabilities. The proportion of CVD deaths relative to all deaths derived from GBD was applied to the UN all-cause mortality estimates to obtain annual CVD and non-CVD mortality probabilities, denoted as $m_{CVD_{a,t}} and m_{nonCVD_{a,t}}$, where *a* is the age of the individual and *t* is calendar year.

**Step 2: Estimating state-specific hazard ratios**.

We then used CLHLS (2002–2005 and 2014–2018), which includes cause-of-death information, to estimate HRs for CVD and non-CVD mortality across health states. For CVD mortality, two Cox models were fitted:

$HR\left( t,CVD death \right)=h_{0}(t)\exp( age_{35}+sex+age_{35}*sex)$ (11)

$HR\left( t,CVD death \right)= h_{0}(t)\exp(age_{35}+sex+age_{35}*sex+state_{i})$ (12)

Where $age_{35}$ were individual age centred at 35 years old, *t* is the survival time and $state_{i}$ was ${State}_{1-8}$.

Formula (11) to yields sex-specific baseline TPs (regardless prior health state) for single year of age, noted as $PCV{Ddeath}_{a}$, and formula (12) yield state- and sex-specific TPs for single year of age, noted as ${PCVDdeath}_{a,i}.$ The ratio

$CVDratio_{a,i}$ = $PCV{Ddeath}_{a,i}$/$PCV{Ddeath}_{a}$

captures how mortality risk differs by health state. Analogous ratios were computed for non-CVD mortality.

**Step 3: Deriving state-specific population mortality.**

Because $m_{CVD_{a,t}}$reflects population-level mortality for China, we replaced the CLHLS baseline probability$PCV{Ddeath}_{a}$with$m_{CVD_{a,t}}$, by applying the state-specific ratio $CVDratio_{a,i}$ to obtain state-specific population CVD mortality, as

$m_{CVD_{a,t,i}}$=$m_{CVD_{a,t}}$ x $CVDratio_{a,i}$

and similarly for non-CVD mortality. This produced state-, age-, sex-, and year-specific TPs for CVD and non-CVD death for all eight health states.

**2.5.3 Calendar effect for CVD and dementia**

The calendar effect of CVD mortality was calculated by ${}_{a,t+1}=\frac{m_{CVD_{a,t+1}}}{m_{CVD_{a,t}}}$, where $m_{CVD_{a,t}}$ is the age-specific probability of death from CVD causes in year $t$. ${}_{a,t+1}$ is an age-specific adjustment factor describing how different the probability of CVD death in year $t+1$ is from the probability of CVD death in the previous year $t$. We assumed that the annual percentage change in CVD incidence was equal to zero, i.e., a constant annual incidence rate. As a sensitivity analysis (S8 & S9 Figs), we further assumed that the annual percentage change in CVD incidence equaled the annual percentage change in CVD mortality. Accordingly, to obtain the incidence of CVD allowing for a calendar effect, we multiplied $P\left( CVD \right) by {}_{a,t+1}$.

In the review of the literature, we assumed three potential future scenarios for the currently increasing dementia incidence: continuous upward, flat or downward trends. Under the flat trend scenario, we assumed a flat trend scenario with constant age-specific incidence (i.e. 0% annual change) to align with prior projections (21), This scenario was used as the main analysis. As sensitivity analyses, we further assumed two alternative calendar effects for dementia incidence: an upward trend and a downward trend. Under the upward trend scenario, we assumed that the dementia incidence would follow a relative annual increase of 2·9%, as an upper limit. The assumption is based on the latest evidence from Shanghai Ageing Studies (22). The calendar effect of dementia was assumed to increase by 2.9% per year (i.e. $P\left( CI \right)_{a,t+1}=P\left( CI \right)_{a,t}* 1.029$). Likewise, we assume an annual 2.9% increase for the TPs related to dementia incidence. Two alternative scenarios were evaluated against this upward trend scenario, and a downward trend with a relative annual decline of 1·0% to reflect the potential impact of public health interventions**(23)**.

2. 6 Uncertainty Interval of the Estimates

The confidence interval of uncertainty was based on the Monte Carlo simulation. Basic Monte Carlo simulation defined ${}_{jm}$ as a vector for each age and calendar-specific value for the input parameter $j$ at iteration $m$ and ${}_{im}$ as a vector for each age and calendar-specific values for the output $i$ at iteration $m$. So, all the age and calendar-specific inputs used in the IMPACT-CAM were defined as $I_{m}$ and outputs estimated by the IMPACT-CAM were defined as $O_{m}$.

For iterations 1 to M, ${}_{j}$ in each iteration was sampled based on the appropriate probability of each input parameter, where prevalence and incidence followed normal distribution and mortality followed uniform distribution. $I_{m}$ based on ${}_{j}$ of each iteration was used in IMPACT-CAM and calculated as $O_{m}$, which included mean, median, 2.5th and 97.5th percentiles of the distribution as uncertainty intervals. 1000 times of iterative sampling from specified distributions for model input parameters is chosen to catch the range of uncertain values for each input parameter (24). Results are stable if we change the iteration times to 2000 or more.

3 Literature review on relative risks of abdominal obesity on dementia, CVD and functional impairment

We used the following PubMed search strategy to identify studies on abdominal obesity and the incidence of dementia, CVD, and functional impairment (FI), as well as recovery from FI. Relative risks (RRs) from Chinese cohorts, together with corresponding RRs and the transition probabilities (TPs) they inform, are summarized in Table S3.

*Dementia:*

**abdominal obesity**[Title/Abstract] OR **central obesity**[Title/Abstract] OR **waist circumference**[Title/Abstract]
AND (**cognitive impairment**[Title/Abstract] OR **mild cognitive impairment**[Title/Abstract] OR **dementia**[Title/Abstract] OR **Alzheimer’s disease**[Title/Abstract])
AND (**cohort**[Title/Abstract] OR **longitudinal**[Title/Abstract] OR **prospective**[Title/Abstract] OR **follow-up**[Title/Abstract] OR **clinical trial**[Title/Abstract])
AND (**risk**[Title/Abstract] OR **incidence**[Title/Abstract] OR **hazard ratio**[Title/Abstract] OR **odds ratio**[Title/Abstract])
AND (China[Mesh] OR **China**[Title/Abstract] OR **Chinese**[Title/Abstract])
AND Humans[Mesh]

*FI:*

**abdominal obesity**[Title/Abstract] OR **central obesity**[Title/Abstract] OR **waist circumference**[Title/Abstract]
AND (**functional impairment**[Title/Abstract] OR **activities of daily living**[Title/Abstract] OR **basic activities of daily living**[Title/Abstract] OR **ADL**[Title/Abstract] OR **disability**[Title/Abstract] OR **frailty**[Title/Abstract])
AND (**cohort**[Title/Abstract] OR **longitudinal**[Title/Abstract] OR **prospective**[Title/Abstract] OR **follow-up**[Title/Abstract] OR **clinical trial**[Title/Abstract])
AND (**risk**[Title/Abstract] OR **incidence**[Title/Abstract] OR **hazard ratio**[Title/Abstract] OR **odds ratio**[Title/Abstract] OR **recovery**[Title/Abstract])
AND (China[Mesh] OR **China**[Title/Abstract] OR **Chinese**[Title/Abstract])
AND Humans[Mesh]

*CVD:*

**abdominal obesity**[Title/Abstract] OR **central obesity**[Title/Abstract] OR **waist circumference**[Title/Abstract]
AND (**cardiovascular disease**[Title/Abstract] OR **CVD**[Title/Abstract] OR **coronary heart disease**[Title/Abstract] OR **stroke**[Title/Abstract] OR **myocardial infarction**[Title/Abstract])
AND (**cohort**[Title/Abstract] OR **longitudinal**[Title/Abstract] OR **prospective**[Title/Abstract] OR **follow-up**[Title/Abstract] OR **clinical trial**[Title/Abstract])
AND (**risk**[Title/Abstract] OR **incidence**[Title/Abstract] OR **hazard ratio**[Title/Abstract] OR **odds ratio**[Title/Abstract])
AND (China[Mesh] OR **China**[Title/Abstract] OR **Chinese**[Title/Abstract])
AND Humans[Mesh]

4 Adjustment of Transition Probability

For each abdominal obesity related transition (Table S2), we assumed that the annual transition probability is proportional to the underlying disease incidence in each age–sex stratum. Let $P_{a,s,t}$ denote the prevalence of abdominal obesity in year t for age group a and sex s under the persistent scenario, and$P_{a,s,t}^{'}$ the corresponding prevalence under a counterfactual scenario (improved or optimal). For a binary exposure with relative risk RR for a given outcome, the expected incidence under the persistent scenario can be written as:

$$I_{a,s,t}=I_{0,a,s,t}\times\left[ 1+P_{a,s,t}\left( RR-1 \right) \right],$$

and under the counterfactual scenario as:

$$I_{a,s,t}^{'}=I_{0,a,s,t}\times\left[ 1+P_{a,s,t}^{'}\left( RR-1 \right) \right],$$

Where$I_{0,a,s,t}$ is the incidence in the unexposed group. The Population Impact Fraction (PIF) is then:

$$\mathrm{PI}F_{a,s,t}=\frac{I_{a,s,t}-I_{a,s,t}^{'}}{I_{a,s,t}}=\frac{\left( P_{a,s,t}-P_{a,s,t}^{'} \right)\left( RR-1 \right)}{1+P_{a,s,t}\left( RR-1 \right)}.$$

By definition, $\mathrm{PI}F_{a,s,t}$represents the proportional reduction in incidence associated with the change in abdominal obesity prevalence. We applied the same proportional change to the baseline transition probability for the persistent scenario, so that the scenario-specific transition probability is given by:

$$TP_{a,s,t}^{\mathrm{scenario}}=TP_{a,s,t}^{\mathrm{persistent}}\times\left( 1-PIF_{a,s,t} \right).$$

Thus, when abdominal obesity prevalence is reduced$\left( P_{a,s,t}^{'}<P_{a,s,t} \right),PIF_{a,s,t}>0$and the corresponding transition probability is proportionally reduced. This calculation was performed separately for each age group, sex, year (2020–2050) and for each of the three scenarios.

5 Age-stratified results

The IMPACT-CAM model estimated age-specific changes in disease burden (relative to the persistent scenario). To illustrate differences in prevalence across age groups, the main analysis results were stratified into 5-year age bands (S6 & S7 Figs).

6 Sensitivity analysis for different calendar effect

The IMPACT model simulated alternative calendar effects for dementia and CVD incidence. In the main analysis, we assumed no calendar effect for either dementia or CVD. Simulations incorporating other calendar effect assumptions were also conducted, with results shown as changes in the total number of cases (S8 Fig) and per 100 000 population (S9 Fig).

References

1. Popkin BM, Du SF, Zhai FY, Zhang B. Cohort Profile: The China Health and Nutrition Survey-monitoring and understanding socio-economic and health change in China, 1989-2011. International Journal of Epidemiology. 2010;39(6):1435-40.

2. Zhao YH, Hu YS, Smith JP, Strauss J, Yang GH. Cohort Profile: The China Health and Retirement Longitudinal Study (CHARLS). International Journal of Epidemiology. 2014;43(1):61-8.

3. Gu D, Feng Q, Chen H, Zeng Y. Chinese Longitudinal Healthy Longevity Survey (CLHLS). In: Gu D, Dupre ME, editors. Encyclopedia of Gerontology and Population Aging. Cham: Springer International Publishing; 2020. p. 1-14.

4. United Nations DoEaSA, Population Division (2022). World Population Prospects 2022 [Available from: <https://population.un.org/wpp2022/>.

5. GBoDC N. Global Burden of Disease Study 2019 (GBD 2019) Results 2024 [Available from: <https://vizhub.healthdata.org/gbd-results/>.

6. Ahmadi-Abhari S, Guzman-Castillo M, Bandosz P, Shipley MJ, Muniz-Terrera G, Singh-Manoux A, et al. Temporal trend in dementia incidence since 2002 and projections for prevalence in England and Wales to 2040: modelling study. Bmj-Brit Med J. 2017;358.

7. Guzman-Castillo M, Ahmadi-Abhari S, Bandosz P, Capewell S, Steptoe A, Singh-Manoux A, et al. Forecasted trends in disability and life expectancy in England and Wales up to 2025: a modelling study. Lancet Public Health. 2017;2(7):e307-e13.

8. Liu YY, Wu YJ, Cai JH, Huang Y, Chen YT, Venkatraman TM, et al. Is there a common latent cognitive construct for dementia estimation across two Chinese cohorts? Alzheimer's & Dementia: Diagnosis, Assessment & Disease Monitoring. 2022;14(1).

9. Chertkow H, Nasreddine Z, Joanette Y, Drolet V, Kirk J, Massoud F, et al. Mild cognitive impairment and cognitive impairment, no dementia: Part A, concept and diagnosis. Alzheimers Dement. 2007;3(4):266-82.

10. Ma F. Diagnostic and Statistical Manual of Mental Disorders-5 (DSM-5). In: Gu D, Dupre ME, editors. Encyclopedia of Gerontology and Population Aging. Cham: Springer International Publishing; 2021. p. 1414-25.

11. Katz S, Downs TD, Cash HR, Grotz RC. Progress in development of the index of ADL. Gerontologist. 1970;10(1):20-30.

12. Zhang X, Dupre ME, Qiu L, Zhou W, Zhao Y, Gu D. Urban-rural differences in the association between access to healthcare and health outcomes among older adults in China. BMC Geriatr. 2017;17(1):151.

13. Xu W, Li YX, Hu Y, Wu C. Association of Frailty with recovery from disability among community-dwelling Chinese older adults: China health and retirement longitudinal study. BMC Geriatr. 2020;20(1):119.

14. Zhou M, Wang H, Zeng X, Yin P, Zhu J, Chen W, et al. Mortality, morbidity, and risk factors in China and its provinces, 1990-2017: a systematic analysis for the Global Burden of Disease Study 2017. Lancet. 2019;394(10204):1145-58.

15. Curran PJ, Hussong AM, Cai L, Huang W, Chassin L, Sher KJ, et al. Pooling data from multiple longitudinal studies: the role of item response theory in integrative data analysis. Dev Psychol. 2008;44(2):365-80.

16. Allen J, Inder KJ, Lewin TJ, Attia JR, Kay-Lambkin FJ, Baker AL, et al. Integrating and extending cohort studies: lessons from the eXtending Treatments, Education and Networks in Depression (xTEND) study. BMC Med Res Methodol. 2013;13:122.

17. Roth GA, Mensah GA, Johnson CO, Addolorato G, Ammirati E, Baddour LM, et al. Global Burden of Cardiovascular Diseases and Risk Factors, 1990-2019: Update From the GBD 2019 Study. J Am Coll Cardiol. 2020;76(25):2982-3021.

18. Chen H, Liu Y, Wu X, Qiao X. A Comparative Study on the Level of Disability among Older People in China: Based on the Data from 4 National Surveys. South China Population. 2021;36(167):1-12.

19. Qi S, Yin P, Zhang H, Zhang Q, Xiao Y, Deng Y, et al. Prevalence of Dementia in China in 2015: A Nationwide Community-Based Study. Front Public Health. 2021;9:733314.

20. Briggs A, Sculpher M. An introduction to Markov modelling for economic evaluation. Pharmacoeconomics. 1998;13(4):397-409.

21. Estimation of the global prevalence of dementia in 2019 and forecasted prevalence in 2050: an analysis for the Global Burden of Disease Study 2019. Lancet Public Health. 2022;7(2):e105-e25.

22. Ding D, Zhao QH, Wu WQ, Xiao ZX, Liang XN, Luo JF, et al. Prevalence and incidence of dementia in an older Chinese population over two decades: The role of education. Alzheimers Dement. 2020;16(12):1650-62.

23. Li F, Qin W, Zhu M, Jia J. Model-Based Projection of Dementia Prevalence in China and Worldwide: 2020-2050. J Alzheimers Dis. 2021;82(4):1823-31.

24. Liu Y, Wu Y, Chen Y, Lobanov-Rostovsky S, Liu Y, Zeng M, et al. Projection for dementia burden in China to 2050: a macro-simulation study by scenarios of dementia incidence trends. The Lancet Regional Health – Western Pacific. 2024;50.
