# Supplementary material for: Impact of abdominal obesity prevalence trends on dementia, cardiovascular disease, functional impairment, and mortality in older Chinese adults: A Markov scenario simulation, 2020–2050
Source: PLoS Med. 2026 Apr 7;23(4):e1004970. doi: 10.1371/journal.pmed.1004970 (PMC13082697; doi:10.1371/journal.pmed.1004970)
Supplement: S1 Table — Note: IMPACT-CAM, IMPACT-Chinese Ageing Model. (DOCX) [file pmed.1004970.s011.docx]

**Table S1** Summary of assumptions underlying the IMPACT-CAM model

| **Assumption** | **Explanation/Evidence** |
| --- | --- |
| IMPACT-CAM model is a probabilistic Markov model simulating the health transitions to death in the Chinese population of age 35+. The input data are age- and sex-specific prevalence, and the transition probabilities (TPs) by age, sex and calendar year. | |
| **Population numbers by age and sex** | |
| Baseline population aged 35–100, and future population aged 35 were obtained from the UN (2022). **Assumption 1:** The UN's population projection for China is realistic. | UN (2022) is the latest population projection of China based on the 2020 China Census, reports and surveys by China's national statistical authorities up to 2021(1) . |
| **Projection baseline prevalence** | |
| Age- and sex-specific prevalence of health states at the model baseline were obtained from the China Health Aging and Retirement Longitudinal Study (CHARLS). **Assumption 2:** CHARLS is a representative nationwide ageing survey of China. | In 2011, CHARLS enlisted participants aged 45 years and above via multi-tiered random sampling across China, with the response rate exceeding 85% in biennial follow-up waves (2013, 2015 and 2018). Refreshment cohorts for those aged less than 50 years are periodically introduced to the study. The representativeness of CHARLS was maintained by low attrition rates and weighting. |
| Four waves of CHARLS data were pooled to estimate the prevalence of health status of the mid-point of the cohort, which is 2015 (projection baseline). This pooling of data was undertaken to enhance statistical power. **Assumption 3:** The pooled data from all waves of CHARLS yields adequate estimates of health state prevalence at the survey's midpoint. | The prevalence of each health status (i.e. cardiovascular diseases (CVD), functional impairment (FI), and dementia) exhibits a linear trend throughout the follow-up waves. The estimated prevalence derived from the pooled CHARLS data aligned with the prevalence values procured at the mid-point of CHARLS (2015) as well as external data, demonstrating a good representation of the Chinese older population. |
| **Assumption 4:** The number of individuals in each state is calculated by adding the number of individuals in that state from the previous year and the number of new cases that occurred, and subtracting those who transitioned to another health state or death state according to the transition probabilities (TPs). | Epidemiological concept of Markov models. |
| **Transition probability (TP)** | |
| TPs were obtained as a function of age and sex from incident cases between wave n and n+ 1 of CHARLS, based on pooled data attributing to the survey midpoint. **Assumption 5:** The age- and sex-specific TPs (equivalent to incidence) of CVD, FI, and cognitive impairment (CI) of CHARLS are similar to those of the whole Chinese population. | TPs are based on representative populations with sampling weights, providing universal estimates for age and sex combinations, encompassing the combined effects of various variables, including education, marital status and region. TPs or incidence of CVD, FI and CI by age and sex were comparable with age- and sex-specific incidence values obtained from external evidence in the mid-point time. |
| **Assumption 6:** CVD and non-CVD mortality of the Chinese Longitudinal Healthy Longevity Study (CLHLS) were similar to those of the entire Chinese population from the National Statistical Bureau. | [Cause-specific deaths were provided by CLHLS 2002–2005 and 2014–2018. CLHLS is a nationwide ageing cohort with an adequate response rate. Survey weight was applied to ensure population representativeness. Deaths predicted by IMPACT-CAM matched with Global Burden of Diseases (GBD) estimates](https://pmc.ncbi.nlm.nih.gov/articles/PMC11342197/table/tbl1/#appsec1)  **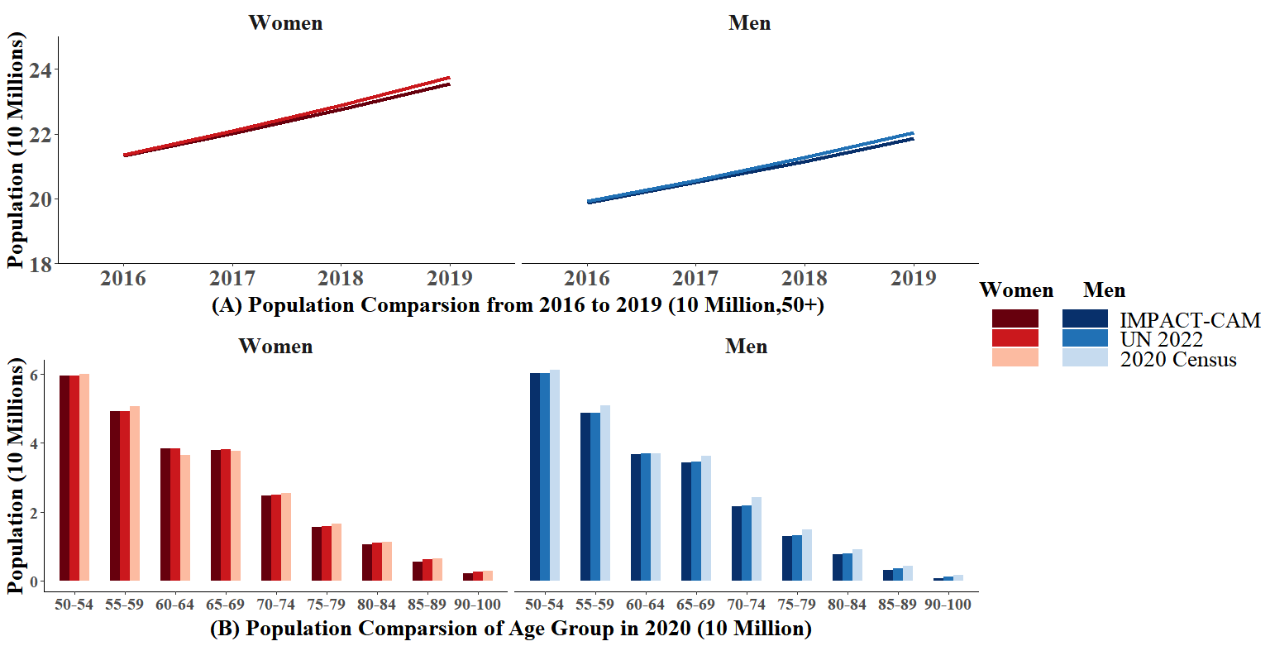** |
| **Assumption 7:** TPs are considered as a weighted average of the different levels of severity of each disease. Similarly, the survival of people in each health state is assumed to be equivalent to the weighted average survival of people with different severities. | Under the assumption that CHARLS and CLHLS are both population-representative, the observed severity spectrum of conditions (like CVD, or CI) should be proportionate to that of the population. TPs extracted from two cohorts thus represent a weighted average of the severity spectrum of health conditions. Multiplying the weighted average TP by the total number of individuals in a given health state is mathematically equal to the sum of the individual products of severity-specific TPs and the respective population sizes in that health state. |
| **Assumption 8:** The model considered the effect of comorbidities such as hypertension, diabetes and BMI et al. | Estimates for risks of dementia, CVD, FI and death of CHARLS and CLHLS represent a weighted average of risk levels across the spectrum of severity of these conditions and their comorbidities. |
| **Calendar trend** | |
| TPs (mortality rates and incidence of CVD and dementia) change over time | |
| **Assumption 9:** The downward trends in mortality observed over the past will continue, and life expectancy is expected to increase. | GBD estimates show that CVD and non-CVD mortality rates followed a decline consistently from 1990 to 2019. We assumed this trend would be most likely to continue and applied the cause-specific ratio of CVD/Non-CVD estimated from GBD (1990–2019) to the UN's mortality projection from 2020 to 2050  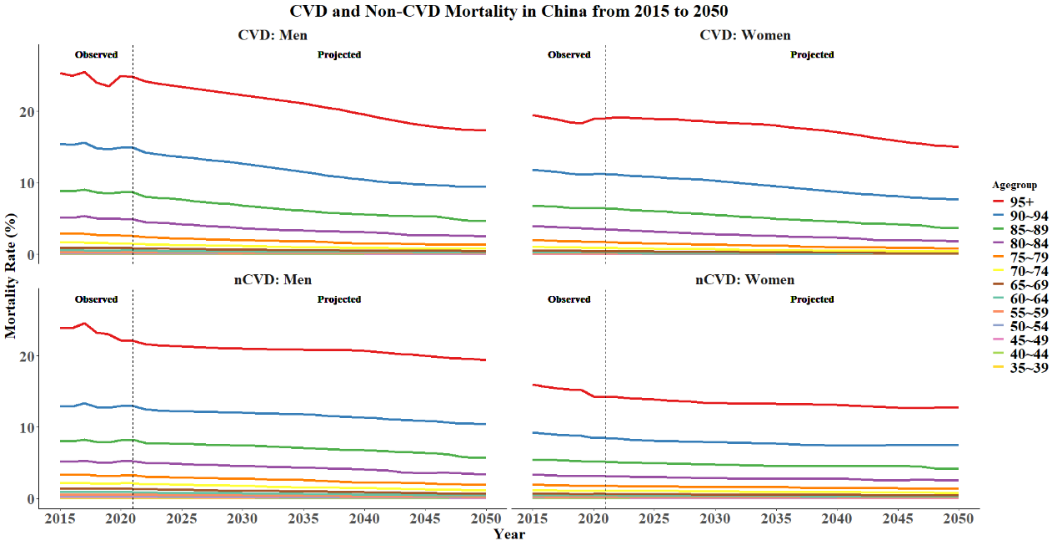  Along with the reduction in mortality rates, we projected a subsequent increase in life expectancy. To examine the uncertainty of this assumption, we conducted a sensitivity analysis assuming a constant mortality rate from 2022.  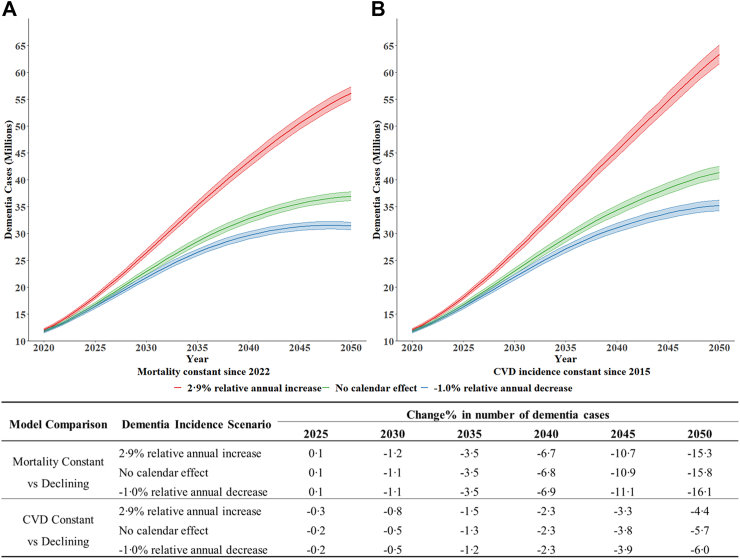 |
| **Assumption 10:** The trends in CVD incidence over time align with the cardiovascular mortality trend. | In line with IMPACT-BAM (2),we assumed the calendar effect of CVD incidence would decline in parallel with the future calendar effect of mortality. Sensitivity analysis was conducted assuming CVD incidence would remain stable after 2015. |
| **Assumption 11:** Trends in incidence of CI and dementia would follow three possible future scenarios: upward, flat or downward. | Under the upward trend scenario, we assumed that the dementia incidence would follow a relative annual increase of 2.9%, as an upper limit (3). A flat trend scenario with constant age-specific incidence (i.e. 0% annual change) to align with prior projections; and a downward trend with a relative annual decline of 1.0% to reflect the potential impact of public health interventions. |
| **Assumption 12:** The incidence of functional impairment remains constant over the projection | Given prior studies have shown a stable trend in the incidence of FI in China (4), no calendar effect of FI was assumed. |
| **Assumption 13:** Survival with CVD, dementia, or FI changes in proportion to changes in overall life expectancy. | IMPACT-CAM considers that survival from each health state is indirectly influenced by changes in mortality rates. It is assumed that the ratio of mortality rates for each health state of the model, compared to the general population, is similar to that observed in CHARLS and CLHLS. Therefore, the model's measures of mortality for each health state change are similar with that observed in the overall population. |
| **Competing risk** | |
| **Assumption 14:** Deaths due to CVD and non-CVD (cancer etc.) and changes in cause-specific mortality are competing risks for the development of dementia cases. | In the context of the model, CVD and non-CVD constitute the terminal states. If an individual die from any cause, they are no longer at risk of developing dementia. |

**References**

1. United Nations DoEaSA, Population Division (2022). World Population Prospects 2022 [Available from: <https://population.un.org/wpp2022/>.

2. Ahmadi-Abhari S, Guzman-Castillo M, Bandosz P, Shipley MJ, Muniz-Terrera G, Singh-Manoux A, et al. Temporal trend in dementia incidence since 2002 and projections for prevalence in England and Wales to 2040: modelling study. Bmj-Brit Med J. 2017;358.

3. Yixin L, Marzieh A, Jing L, Archana S-M, Eric B. P30 Trend in dementia incidence in China 2002–2014: population-based longitudinal study. Journal of Epidemiology and Community Health. 2021;75(Suppl 1):A56.

4. Yan M, Qin T, Yin P. Disabilities in activities of daily living and in instrumental activities of daily living among older adults in China, 2011&#x2013;15: a longitudinal cohort study. The Lancet. 2019;394:S82.
