# Supplementary material for: Impact of abdominal obesity prevalence trends on dementia, cardiovascular disease, functional impairment, and mortality in older Chinese adults: A Markov scenario simulation, 2020–2050
Source: PLoS Med. 2026 Apr 7;23(4):e1004970. doi: 10.1371/journal.pmed.1004970 (PMC13082697; doi:10.1371/journal.pmed.1004970)
Supplement: S2 Table — Notes: CVD, cardiovascular diseases; FI, functional impairment; CI, Cognitive Impairment; CIND, Cognitive impairment no dementia; Dis-free, free of CVD, CI, FI or Dementia. (DOCX) [file pmed.1004970.s012.docx]

**S2 Table Input Baseline Prevalence (%) at 2015 of IMPACT-CAM**

| Gender | Age Range | Dis-free | CVD only | CVD & CI | CIND | CVD & FI | CVD & CI & FI | CI & FI | FI only |
| --- | --- | --- | --- | --- | --- | --- | --- | --- | --- |
| Men | 35-39 | 94.65 (94.63~94.67) | 4.89 (4.87~4.91) | 0.00 (0.00~0.00) | 0.00 (0.00~0.00) | 0.02 (0.02~0.02) | 0.00 (0.00~0.00) | 0.00 (0.00~0.00) | 0.44 (0.44~0.45) |
| Men | 40-44 | 93.08 (93.06~93.11) | 6.28 (6.26~6.30) | 0.00 (0.00~0.00) | 0.00 (0.00~0.00) | 0.04 (0.04~0.04) | 0.00 (0.00~0.00) | 0.00 (0.00~0.00) | 0.59 (0.59~0.60) |
| Men | 45-49 | 91.20 (91.17~91.22) | 7.96 (7.93~7.98) | 0.00 (0.00~0.00) | 0.00 (0.00~0.00) | 0.07 (0.07~0.07) | 0.00 (0.00~0.00) | 0.00 (0.00~0.00) | 0.78 (0.77~0.78) |
| Men | 50-54 | 86.04 (86.02~86.07) | 9.67 (9.65~9.70) | 0.26 (0.26~0.27) | 2.35 (2.34~2.37) | 0.11 (0.11~0.12) | 0.05 (0.05~0.06) | 0.48 (0.48~0.49) | 1.01 (1.00~1.02) |
| Men | 55-59 | 82.20 (82.17~82.22) | 12.31 (12.29~12.33) | 0.37 (0.37~0.37) | 2.48 (2.47~2.49) | 0.20 (0.20~0.20) | 0.14 (0.14~0.14) | 0.94 (0.93~0.95) | 1.35 (1.34~1.36) |
| Men | 60-64 | 77.89 (77.87~77.92) | 14.99 (14.97~15.01) | 0.51 (0.51~0.52) | 2.66 (2.65~2.68) | 0.33 (0.33~0.33) | 0.31 (0.31~0.31) | 1.58 (1.57~1.59) | 1.72 (1.71~1.73) |
| Men | 65-69 | 72.37 (72.34~72.40) | 18.09 (18.07~18.11) | 0.74 (0.74~0.74) | 2.94 (2.93~2.95) | 0.55 (0.55~0.55) | 0.63 (0.63~0.63) | 2.49 (2.48~2.50) | 2.20 (2.19~2.20) |
| Men | 70-74 | 65.43 (65.40~65.47) | 21.38 (21.35~21.41) | 1.09 (1.09~1.10) | 3.33 (3.32~3.34) | 0.91 (0.91~0.92) | 1.26 (1.25~1.27) | 3.82 (3.80~3.83) | 2.78 (2.77~2.79) |
| Men | 75-79 | 57.15 (57.09~57.20) | 24.30 (24.25~24.35) | 1.64 (1.63~1.65) | 3.83 (3.82~3.85) | 1.48 (1.47~1.48) | 2.45 (2.44~2.46) | 5.71 (5.68~5.73) | 3.44 (3.43~3.46) |
| Men | 80-84 | 47.80 (47.72~47.86) | 26.25 (26.19~26.30) | 2.47 (2.46~2.49) | 4.47 (4.44~4.49) | 2.31 (2.29~2.32) | 4.47 (4.45~4.49) | 8.07 (8.03~8.10) | 4.17 (4.15~4.19) |
| Men | 85-89 | 37.60 (37.51~37.69) | 26.58 (26.50~26.65) | 3.75 (3.72~3.78) | 5.26 (5.22~5.30) | 3.52 (3.50~3.55) | 7.64 (7.60~7.69) | 10.70 (10.65~10.76) | 4.94 (4.91~4.97) |
| Men | 90- | 24.91 (24.79~25.02) | 23.33 (23.21~23.44) | 6.26 (6.19~6.31) | 6.43 (6.37~6.50) | 5.67 (5.63~5.71) | 13.59 (13.52~13.67) | 13.96 (13.88~14.03) | 5.85 (5.81~5.89) |
| Women | 35-39 | 90.45 (90.42~90.48) | 8.92 (8.89~8.95) | 0.00 (0.00~0.00) | 0.00 (0.00~0.00) | 0.06 (0.06~0.06) | 0.00 (0.00~0.00) | 0.00 (0.00~0.00) | 0.58 (0.57~0.58) |
| Women | 40-44 | 88.49 (88.47~88.52) | 10.65 (10.63~10.67) | 0.00 (0.00~0.00) | 0.00 (0.00~0.00) | 0.09 (0.09~0.09) | 0.00 (0.00~0.00) | 0.00 (0.00~0.00) | 0.76 (0.75~0.77) |
| Women | 45-49 | 86.29 (86.27~86.32) | 12.57 (12.55~12.60) | 0.00 (0.00~0.00) | 0.00 (0.00~0.00) | 0.15 (0.14~0.15) | 0.00 (0.00~0.00) | 0.00 (0.00~0.00) | 0.99 (0.98~1.00) |
| Women | 50-54 | 80.00 (79.97~80.03) | 14.08 (14.05~14.10) | 0.55 (0.55~0.55) | 3.12 (3.11~3.14) | 0.23 (0.22~0.23) | 0.11 (0.11~0.11) | 0.63 (0.62~0.63) | 1.28 (1.27~1.29) |
| Women | 55-59 | 76.04 (76.02~76.06) | 16.56 (16.54~16.58) | 0.72 (0.71~0.72) | 3.28 (3.27~3.29) | 0.37 (0.37~0.37) | 0.24 (0.24~0.24) | 1.10 (1.09~1.11) | 1.70 (1.69~1.70) |
| Women | 60-64 | 71.83 (71.80~71.85) | 18.84 (18.82~18.87) | 0.92 (0.92~0.93) | 3.51 (3.50~3.53) | 0.57 (0.56~0.57) | 0.45 (0.45~0.46) | 1.72 (1.71~1.72) | 2.15 (2.14~2.16) |
| Women | 65-69 | 66.63 (66.61~66.66) | 21.24 (21.21~21.26) | 1.24 (1.24~1.24) | 3.88 (3.87~3.89) | 0.88 (0.87~0.88) | 0.82 (0.82~0.83) | 2.57 (2.56~2.58) | 2.74 (2.73~2.75) |
| Women | 70-74 | 60.35 (60.31~60.38) | 23.46 (23.43~23.49) | 1.72 (1.71~1.73) | 4.40 (4.39~4.42) | 1.36 (1.35~1.36) | 1.47 (1.47~1.48) | 3.76 (3.75~3.78) | 3.47 (3.46~3.48) |
| Women | 75-79 | 53.10 (53.06~53.16) | 25.14 (25.10~25.18) | 2.43 (2.42~2.44) | 5.12 (5.10~5.13) | 2.06 (2.06~2.07) | 2.52 (2.51~2.53) | 5.29 (5.27~5.31) | 4.33 (4.32~4.35) |
| Women | 80-84 | 45.22 (45.16~45.27) | 25.89 (25.84~25.94) | 3.47 (3.45~3.49) | 6.03 (6.00~6.05) | 3.05 (3.03~3.06) | 4.04 (4.02~4.05) | 7.01 (6.97~7.03) | 5.30 (5.27~5.32) |
| Women | 85-89 | 36.55 (36.46~36.63) | 25.32 (25.26~25.39) | 5.04 (5.01~5.07) | 7.22 (7.18~7.27) | 4.45 (4.43~4.48) | 6.17 (6.15~6.20) | 8.85 (8.81~8.89) | 6.39 (6.35~6.43) |
| Women | 90- | 25.13 (25.01~25.24) | 21.59 (21.50~21.71) | 8.11 (8.04~8.17) | 9.13 (9.07~9.19) | 6.93 (6.89~6.96) | 10.01 (9.95~10.06) | 11.29 (11.23~11.34) | 7.82 (7.78~7.87) |

Notes: CVD: cardiovascular diseases. FI: functional impairment. CI: Cognitive Impairment. CIND: Cognitive impairment no dementia. Dis-free: free of CVD, CI, FI or Dementia.
