# Supplementary material for: Impact of abdominal obesity prevalence trends on dementia, cardiovascular disease, functional impairment, and mortality in older Chinese adults: A Markov scenario simulation, 2020–2050
Source: PLoS Med. 2026 Apr 7;23(4):e1004970. doi: 10.1371/journal.pmed.1004970 (PMC13082697; doi:10.1371/journal.pmed.1004970)
Supplement: S3 Table — Notes: CVD, cardiovascular diseases; TP, transition probability; RR, relative risk. RR represents the relative risks of each outcome associated with abdominal obesity compared with no abdominal obesity. Estimated from Chinese longitudinal studies, adjusted for key sociodemographic and lifestyle factors but not for potential intermediates in the causal pathway (e.g., hypertension, diabetes). (DOCX) [file pmed.1004970.s013.docx]

**S3 Table** Transition probabilities affected by the change in abdominal obesity

| **Outcome** | **TPs affected** | **RR (95% CI) from literature search** |
| --- | --- | --- |
| CVD mortality | TP _i,9_ (i=1-8) | 1.49 (1.33, 1.66) (1) |
| Non-CVD mortality | TP _i,10_ (i=1-8) | 1.27 (1.07, 1.50) (1) |
| CVD incidence | TP _1,2_  TP _4,3_ | Female：1.45 (1.42, 1.48) (2)  Male： 1.53 (1.49, 1.56) (2) |
| Dementia incidence | TP _1,3_  TP _1,4_  TP _2,3_ | Age 45-65 years  1.45 (1.15, 1.84) (3) |
| Functional impairment incidence | TP _2,5_  TP _3,6_  TP _1,8_  TP _4,7_ | Age ≥80 years  Female：1.10 (0.93, 1.31) (4)  Male： 1.17 (0.95, 1.45) (4) |
| Functional impairment reversal | TP _5,2_  TP _6,3_  TP _8,1_  TP _7,4_ | 0.90 (0.50, 1.60) (5) |

Notes: CVD: cardiovascular diseases. TP: transition probability. RR: relative risk.

RR represents the relative risks of each outcome associated with abdominal obesity compared with no abdominal obesity. Estimated from Chinese longitudinal studies, adjusted for key sociodemographic and lifestyle factors but not for potential intermediates in the causal pathway (e.g., hypertension, diabetes).

**References**

1. Zhu N, Yu C, Guo Y, Bian Z, Han Y, Yang L, et al. Adherence to a healthy lifestyle and all-cause and cause-specific mortality in Chinese adults: a 10-year prospective study of 0.5 million people. Int J Behav Nutr Phys Act. 2019;16(1):98.

2. Li JC, Lyu J, Gao M, Yu CQ, Guo Y, Bian Z, et al. [Association of body mass index and waist circumference with major chronic diseases in Chinese adults]. Chinese Journal of Epidemiology. 2019;40(12):1541-7.

3. Zeng MR, Chen YT, Lobanov-Rostovsky S, Liu YY, Steptoe A, Brunner EJ, et al. Adiposity and dementia among Chinese adults: longitudinal study in the China Health and Retirement Longitudinal Study (CHARLS). International Journal of Obesity. 2025;49(4):706-14.

4. Ju AP, Zhou JH, Gu H, Ye LL, Chen C, Guo YB, et al. [Association of body mass index and waist circumference with frailty among people aged 80 years and older in Chinese]. Chinese Journal of Epidemiology. 2022;56(11):1584-90.

5. Walter S, Kunst A, Mackenbach J, Hofman A, Tiemeier H. Mortality and disability: the effect of overweight and obesity. Int J Obes (Lond). 2009;33(12):1410-8.
