# Supplementary material for: Impact of abdominal obesity prevalence trends on dementia, cardiovascular disease, functional impairment, and mortality in older Chinese adults: A Markov scenario simulation, 2020–2050
Source: PLoS Med. 2026 Apr 7;23(4):e1004970. doi: 10.1371/journal.pmed.1004970 (PMC13082697; doi:10.1371/journal.pmed.1004970)
Supplement: S4 Table — Notes: CVD, cardiovascular diseases; FI, functional impairment. The data in parentheses represent the 95% uncertainty intervals. Persistent scenario: The prevalence of abdominal obesity continues to increase. Optimal scenario: prevalence of abdominal obesity remains unchanged. Improved scenario: growth rate of abdominal obesity prevalence is reduced by 50%. (DOCX) [file pmed.1004970.s014.docx]

**S4 Table Number of incident cases of dementia, disability and CVD avoided (cumulative since 2015) for optimal and improved scenario versus persistent scenario in Chinese population aged ≥ 65 years.**

|  | **Reduction in dementia cases** | | **Reduction in disability cases** | | **Reduction in CVD cases** | |
| --- | --- | --- | --- | --- | --- | --- |
|  | **Cases avoided (thousand)** | **Cases avoided per 100,000 population** | **Cases avoided**  **(thousand)** | **Cases avoided per 100,000 population** | **Cases avoided (thousand)** | **Cases avoided per 100,000 population** |
| **Optimal scenario** | | | | | | |
| **All** |  |  |  |  |  |  |
| 2020 | 2.5 (-8.2, 12.3) | -0.3 (-2.4, 1.6) | 7.2 (-12.1, 27.9) | -0.5 (-4.1, 3.3) | 127.3 (82.5, 170.5) | 21.2 (11.5, 30.4) |
| 2030 | -106.6 (-207.8, -21.4) | -8.8 (-15.1, -3.0) | -208.4 (-379.6, -19.8) | -17.3 (-28.4, -5.8) | 545.5 (37.5, 1026.4) | 16.5 (-14.6, 46.7) |
| 2040 | -478.8 (-845.7, -146.5) | -18.9 (-31.1, -7.6) | -949.0 (-1554.5, -309.7) | -36.3 (-56.6, -15.5) | 626.7 (-1071.7, 2319.5) | -12.9 (-65.1, 38.7) |
| 2050 | -1396.0 (-2293.9, -589.1) | -37.4 (-57.8, -18.2) | -2570.6 (-4024.9, -1081.0) | -62.8 (-96.6, -27.9) | -613 .9 (-4390.8, 3117.3) | -58.4 (-139.2, 21.4) |
| **Men** |  |  |  |  |  |  |
| 2020 | 1.1 (-1.3, 3.8) | 0.2 (-0.9, 1.5) | 2.8 (-2.8, 8.7) | 0.3 (-2.3, 3.0) | 59.0 (47.7, 73.4) | 23.4 (17.6, 30.5) |
| 2030 | -22.6 (-56.8, 15.7) | -4.9 (-10.8, 1.6) | -55.7 (-119.0, 22.2) | -11.8 (-22.2, 0.5) | 339.1 (178.1, 520.1) | 30.5 (6.2, 57.0) |
| 2040 | -136.3 (-299.0, 42.8) | -13.8 (-28.1, 1.8) | -309.6 (-583.7, 9.2) | -28.5 (-51.0, -4.1) | 618.2 (-11.9, 1284.3) | 10.5 (-36.2, 60.1) |
| 2050 | -473.4 (-911.1, 12.0) | -31.9 (-56.8, -5.6) | -911.8 (-1635.3, -140.7) | -53.9 (-91.6, -13.4) | 435.6 (-1065.6, 2039.5) | -28.6 (-104.2, 50.9) |
| **Women** |  |  |  |  |  |  |
| 2020 | 1.2 (-9.4, 10.8) | -0.7 (-4.4, 2.8) | 4.4 (-13.6, 24.4) | -1.2 (-7.5, 5.2) | 68.1 (26.2, 107.2) | 19.2 (2.9, 34.5) |
| 2030 | -85.1 (-176.5, 0.5) | -12.0 (-22.3, -2.3) | -152.0 (-309.1, 17.7) | -22.0 (-40.0, -3.8) | 199.6 (-270.9, 634.9) | 3.3 (-48.1, 52.7) |
| 2040 | -348.7 (-671.6, -32.0) | -23.3 (-42.0, -4.5) | -645.7 (-1205.3, -68.9) | -42.9 (-76.1, -9.2) | -22.1 (-1568.6, 1464.7) | -31.9 (-117.0, 48.4) |
| 2050 | -936.6 (-1705.4, -194.2) | -42.3 (-74.3, -11.3) | -1659.3 (-2992.2, -315.4) | -70.3 (-122.7, -16.1) | -1059.9 (-4477.7, 2179.1) | -82.0 (-211.2, 39.8) |
| **Improved scenario** | | | | | | |
| **All** |  |  |  |  |  |  |
| 2020 | 1.2 (-4.0, 6.8) | -0.2 (-1.1, 0.9) | 3.8 (-5.9, 13.8) | -0.3 (-2.0, 1.6) | 62.5 (40.6, 82.5) | 10.4 (5.6, 14.6) |
| 2030 | -54.8 (-101.2, -6.9) | -4.5 (-7.5, -1.6) | -104.2 (-187.7, -21.2) | -8.6 (-14.3, -3.5) | 257.0 (6.7, 477.8) | 7.2 (-8.2, 20.8) |
| 2040 | -245.1 (-419.6, -77.5) | -9.5 (-15.8, -3.7) | -472.7 (-793.2, -179.0) | -18.0 (-28.4, -8.3) | 254.7 (-576.5, 1008.9) | -7.9 (-35.0, 15.6) |
| 2050 | -711.4 (-1150.3, -294.4) | -18.9 (-29.5, -8.8) | -1289.5 (-2034.2, -569.5) | -31.3 (-48.8, -14.7) | -418.5 (-2365.9, 1250.0) | -31.5 (-74.9, 5.3) |
| **Men** |  |  |  |  |  |  |
| 2020 | 0.7 (-0.5, 2.0) | 0.2 (-0.4, 0.8) | 1.6 (-1.2, 4.5) | 0.2 (-1.1, 1.5) | 29.5 (23.5, 36.7) | 11.7 (8.8, 15.1) |
| 2030 | -11.4 (-28.9, 6.5) | -2.5 (-5.3, 0.5) | -27.2 (-61.0, 7.2) | -5.8 (-10.9, -0.3) | 164.7 (85.2, 252.2) | 14.7 (2.6, 27.7) |
| 2040 | -68.9 (-145.7, 12.9) | -7.1 (-13.7, 0.1) | -151.3 (-286.9, -6.2) | -14.2 (-24.9, -2.8) | 288.4 (-19.0, 615.1) | 4.0 (-19.6, 27.4) |
| 2050 | -241.8 (-442.1, -19.0) | -16.4 (-27.8, -3.8) | -454.9 (-797.6, -96.4) | -27.0 (-45.5, -7.6) | 177.7 (-574.2, 932.1) | -16.6 (-54.6, 20.7) |
| **Women** |  |  |  |  |  |  |
| 2020 | 0.5 (-4.3, 5.7) | -0.4 (-2.1, 1.4) | 2.3 (-6.9, 12.2) | -0.6 (-3.8, 2.6) | 33.1 (12.3, 52.1) | 9.3 (1.1, 16.5) |
| 2030 | -43.0 (-86.4, 2.8) | -6.1 (-10.7, -1.1) | -76.1 (-157.3, -1.7) | -10.9 (-20.0, -2.6) | 97.7 (-135.9, 299.3) | 1.6 (-24.5, 24.2) |
| 2040 | -176.1 (-321.6, -19.0) | -11.8 (-20.6, -2.8) | -320.8 (-601.7, -59.6) | -21.4 (-37.1, -6.2) | -12.9 (-805.0, 666.6) | -16.5 (-61.0, 21.2) |
| 2050 | -473.0 (-814.6, -118.5) | -21.3 (-35.8, -6.2) | -828.1 (-1459.4, -222.1) | -34.8 (-61.7, -9.7) | -550.2 (-2305.7, 955.2) | -41.4 (-107.8, 15.4) |

Notes: CVD: cardiovascular diseases. FI: functional impairment. The data in parentheses represent the 95% uncertainty intervals⸱ Persistent scenario: The prevalence of abdominal obesity continues to increase. Optimal scenario: prevalence of abdominal obesity remains unchanged. Improved scenario: growth rate of abdominal obesity prevalence is reduced by 50%.
