# Supplementary material for: Impact of abdominal obesity prevalence trends on dementia, cardiovascular disease, functional impairment, and mortality in older Chinese adults: A Markov scenario simulation, 2020–2050
Source: PLoS Med. 2026 Apr 7;23(4):e1004970. doi: 10.1371/journal.pmed.1004970 (PMC13082697; doi:10.1371/journal.pmed.1004970)
Supplement: S5 Table — Notes: CVD, cardiovascular diseases; FI, functional impairment. The data in parentheses represent the 95% uncertainty intervals. Persistent scenario: The prevalence of abdominal obesity continues to increase. Optimal scenario: prevalence of abdominal obesity remains unchanged. Improved scenario: growth rate of abdominal obesity prevalence is reduced by 50%. (DOCX) [file pmed.1004970.s015.docx]

**S5 Table Number of deaths avoided (cumulative since 2015) for optimal and improved scenario versus persistent scenario in Chinese population aged ≥ 65 years.**

|  | **Reduction in all-Cause mortality** | | **Reduction in CVD mortality** | | **Reduction in non-CVD mortality** | |
| --- | --- | --- | --- | --- | --- | --- |
|  | **Deaths avoided (thousand)** | **Deaths avoided per 100,000 population** | **Deaths avoided (thousand)** | **Deaths avoided per 100,000 population** | **Deaths avoided (thousand)** | **Deaths avoided per 100,000 population** |
| **Optimal scenario** | | | | | | |
| **All** |  |  |  |  |  |  |
| 2020 | 229.3 (180.5, 277.2) | 39.8 (31.8, 47.9) | 150.8 (118.9, 180.7) | 26.6 (21.0, 31.8) | 78.2 (33.4, 124.7) | 13.1 (5.0, 21.6) |
| 2030 | 1368.4 (1085.2, 1649.8) | 61.7 (48.3, 74.1) | 966.0 (742.4, 1179.2) | 46.5 (34.5, 57.6) | 398.1 (59.3, 749.2) | 15.1 (-2.1, 33.4) |
| 2040 | 3170.6 (2530.7, 3778.9) | 61.3 (48.1, 74.8) | 2443.4 (1799.6, 3048.9) | 55.2 (37.1, 72.2) | 714.9 (-180.8, 1668.3) | 6.7 (-16.5, 31.4) |
| 2050 | 4807.8 (3861.8, 5690.6) | 45.7 (33.2, 58.8) | 4222.7 (2875.5, 5449.6) | 59.6 (32.4, 85.4) | 577.9 (-1087.9, 2354.9) | -13.6 (-43.5, 16.9) |
| **Men** |  |  |  |  |  |  |
| 2020 | 84.4 (54.6, 111.2) | 34.5 (22.7, 45.3) | 50.7 (34.9, 65.2) | 21.1 (14.6, 27.1) | 33.1 (6.9, 61.1) | 13.2 (2.2, 25.0) |
| 2030 | 586.6 (396.3, 764.7) | 63.9 (44.1, 83.2) | 390.1 (268.8, 502.4) | 45.9 (31.6, 61.5) | 191.0 (-15.9, 418.6) | 17.2 (-7.4, 44.3) |
| 2040 | 1413.5 (982.9, 1809.7) | 62.2 (43.4, 80.7) | 1063.9 (713.1, 1417.1) | 57.4 (33.6, 82.3) | 335.9 (-231.7, 936.2) | 4.6 (-28.3, 36.7) |
| 2050 | 2123.8 (1547.5, 2657.9) | 38.5 (22.2, 56.1) | 1928.8 (1187.3, 2680.3) | 63.6 (26.9, 99.8) | 165.8 (-856.4, 1274.4) | -25.8 (-64.1, 13.0) |
| **Women** |  |  |  |  |  |  |
| 2020 | 145.0 (105.3, 188.3) | 44.4 (32.6, 57.0) | 100.3 (72.9, 125.9) | 31.3 (22.8, 39.2) | 45.1 (7.8, 81.9) | 13.2 (1.1, 25.1) |
| 2030 | 779.8 (576.4, 1007.6) | 60.1 (43.1, 76.5) | 577.5 (398.3, 750.0) | 46.8 (29.4, 64.3) | 207.3 (-64.9, 467.9) | 13.1 (-11.6, 37.2) |
| 2040 | 1756.5 (1289.0, 2266.2) | 60.4 (42.3, 81.0) | 1374.7 (887.5, 1851.0) | 52.4 (27.5, 77.2) | 391.8 (-348.4, 1109.5) | 8.4 (-26.3, 41.5) |
| 2050 | 2677.2 (1966.6, 3477.5) | 51.2 (33.2, 72.4) | 2287.9 (1272.1, 3267.6) | 55.4 (17.8, 93.4) | 387.6 (-980.6, 1769.3) | -4.5 (-47.0, 38.7) |
| **Improved scenario** | | | | | | |
| **All** |  |  |  |  |  |  |
| 2020 | 115.8 (90.2, 140.4) | 20.1 (15.8, 24.3) | 75.9 (61.0, 90.5) | 13.4 (10.7, 16.0) | 39.0 (16.0, 62.7) | 6.6 (2.4, 10.9) |
| 2030 | 686.8 (541.9, 826.9) | 30.8 (24.2, 37.1) | 487.4 (376.6, 589.8) | 23.3 (17.7, 29.1) | 197.8 (23.0, 374.3) | 7.5 (-1.8, 16.7) |
| 2040 | 1584.4 (1271.0, 1894.9) | 30.3 (23.8, 37.0) | 1225.8 (932.9, 1512.9) | 27.4 (19.0, 35.9) | 355.1 (-117.9, 833.8) | 2.9 (-9.4, 15.2) |
| 2050 | 2382.5 (1924.2, 2834.9) | 22.0 (15.6, 29.0) | 2109.6 (1471.4, 2721.9) | 29.3 (16.2, 42.8) | 274.6 (-610.8, 1151.4) | -7.2 (-21.8, 7.5) |
| **Men** |  |  |  |  |  |  |
| 2020 | 42.7 (28.4, 55.2) | 17.5 (11.7, 22.4) | 25.2 (18.3, 32.5) | 10.5 (7.6, 13.5) | 17.2 (3.9, 30.1) | 6.9 (1.3, 12.4) |
| 2030 | 297.1 (203.1, 380.1) | 32.2 (22.5, 41.4) | 194.3 (140.5, 255.2) | 23.0 (16.0, 31.1) | 100.1 (-4.9, 203.7) | 9.1 (-3.3, 21.2) |
| 2040 | 709.5 (504.5, 893.4) | 31.0 (22.1, 39.8) | 526.6 (350.3, 713.0) | 28.0 (16.6, 40.3) | 176.6 (-110.3, 452.4) | 2.5 (-13.2, 18.4) |
| 2050 | 1056.9 (781.1, 1310.3) | 18.7 (10.6, 26.7) | 945.4 (573.0, 1342.6) | 30.4 (13.6, 50.6) | 107.7 (-417.2, 610.1) | -12.5 (-30.7, 5.8) |
| **Women** |  |  |  |  |  |  |
| 2020 | 73.0 (52.4, 93.5) | 22.3 (16.0, 28.4) | 50.8 (37.4, 63.3) | 15.8 (11.5, 19.8) | 22.4 (3.1, 40.6) | 6.5 (0.3, 12.4) |
| 2030 | 389.9 (279.4, 499.8) | 29.8 (21.1, 38.5) | 290.7 (199.5, 379.8) | 23.4 (15.0, 31.8) | 100.5 (-38.9, 230.9) | 6.4 (-6.5, 18.6) |
| 2040 | 876.9 (629.9, 1121.1) | 29.9 (21.0, 39.3) | 688.3 (437.2, 936.5) | 26.1 (14.0, 39.3) | 185.1 (-194.1, 536.1) | 3.7 (-13.8, 20.0) |
| 2050 | 1326.9 (954.4, 1693.0) | 24.9 (15.3, 34.7) | 1137.0 (657.5, 1644.9) | 27.4 (10.0, 47.4) | 186.9 (-528.0, 836.7) | -2.5 (-25.8, 17.6) |

Notes: CVD: cardiovascular diseases. FI: functional impairment. The data in parentheses represent the 95% uncertainty intervals⸱ Persistent scenario: The prevalence of abdominal obesity continues to increase. Optimal scenario: prevalence of abdominal obesity remains unchanged. Improved scenario: growth rate of abdominal obesity prevalence is reduced by 50%.
